# Supplementary material for: An innovative hematopoietic stem cell gene therapy approach benefits CLN1 disease in the mouse model
Source: EMBO Mol Med. 2023 Mar 6;15(4):e15968. doi: 10.15252/emmm.202215968 (PMC10086581; doi:10.15252/emmm.202215968)
Supplement: Supplementary file 1 — Appendix [file EMMM-15-e15968-s006.pdf]

Appendix file

An innovative hematopoietic stem cell gene therapy approach benefits CLN1 disease in the mouse model

Marco Peviani, Sabyasachi Das, Janki Patel, Odella Jno-Charles, Rajesh Kumar, Ana Zguro, Tyler D. Mathews, Paolo Cabras, Rita Milazzo, Eleonora Cavalca, Valentina Poletti, Alessandra Biffi.

Table of content

Appendix Fig. S1 .... *1*
Appendix Fig. S2. .... *3*
Appendix Fig. S3. .... *5*
Appendix Fig. S4. .... *7*
Appendix Fig. S5. .... *8*
Appendix Fig. S6 .... *10*
Appendix Table S1. .... *12*
Appendix Table S2. .... *13*
Appendix Table S3. .... *14*
Appendix Table S4. .... *16*
Appendix Table S5. .... *19*
Appendix Table S6. .... *20*
Appendix Table S7. .... *20*
Appendix Table S8. .... *21*
Appendix Table S9. .... *22*

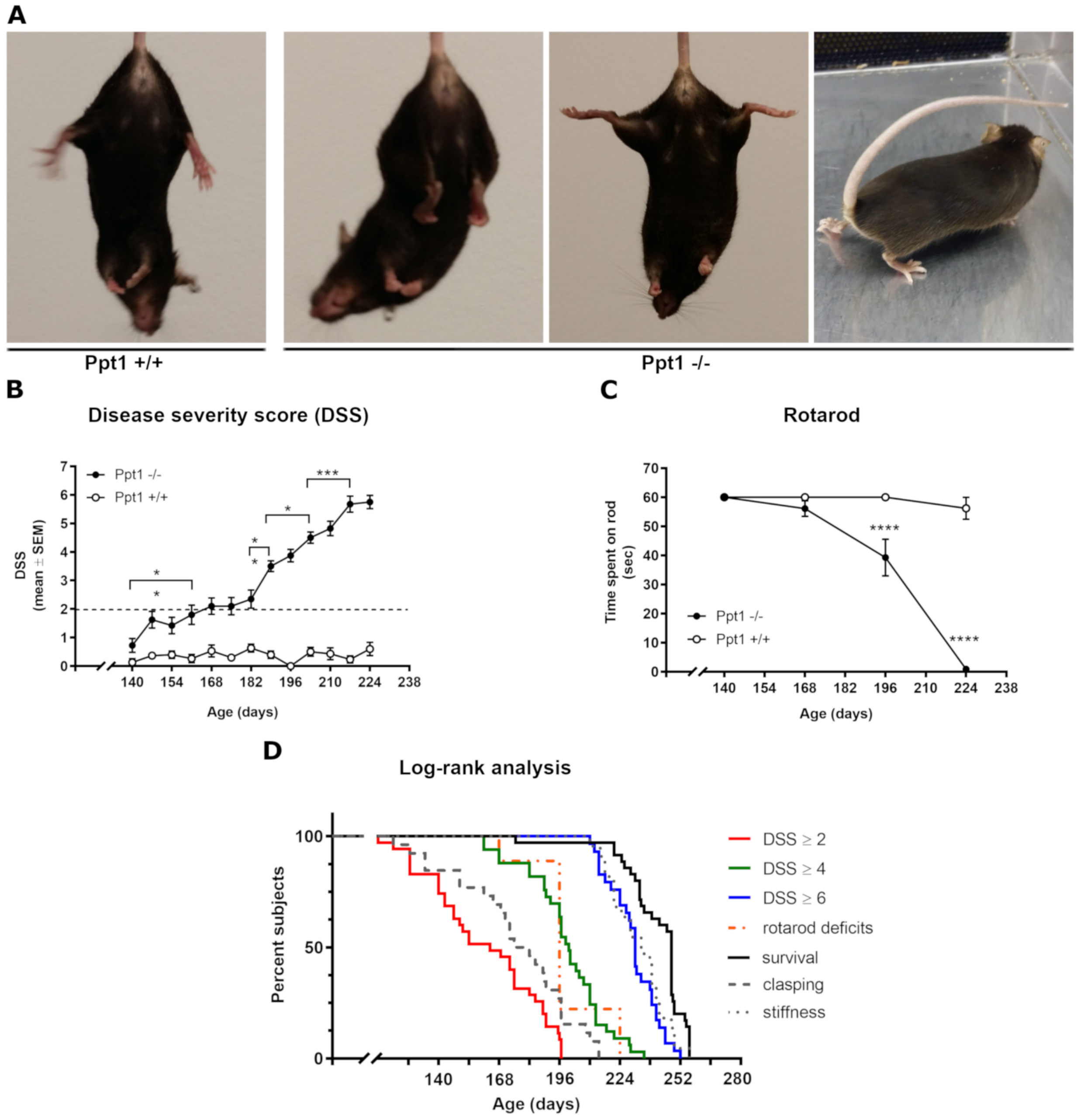

Appendix Fig. S1.

A. Representative pictures of the disease manifestations displayed by symptomatic Ppt1$^{-/-}$ mice during tail suspension. **B.** Progressive deterioration of animals body condition demonstrated by the DSS. * = p< 0.05; ** = p< 0.01; *** = p< 0.001 Repeated Measures ANOVA followed by Tukey's post-hoc test. **C.** Progressive motor deficits in Ppt1$^{-/-}$ mice assessed by rotarod test. ****

= $p < 0.0001$ Repeated Measures ANOVA followed by Tukey's post-hoc test. **D.** Comparison of DSS with different disease manifestations (limbs clasping, stiffness and rotarod deficits) and with survival by Log-rank analysis. See Appendix Table S2 for statistics.

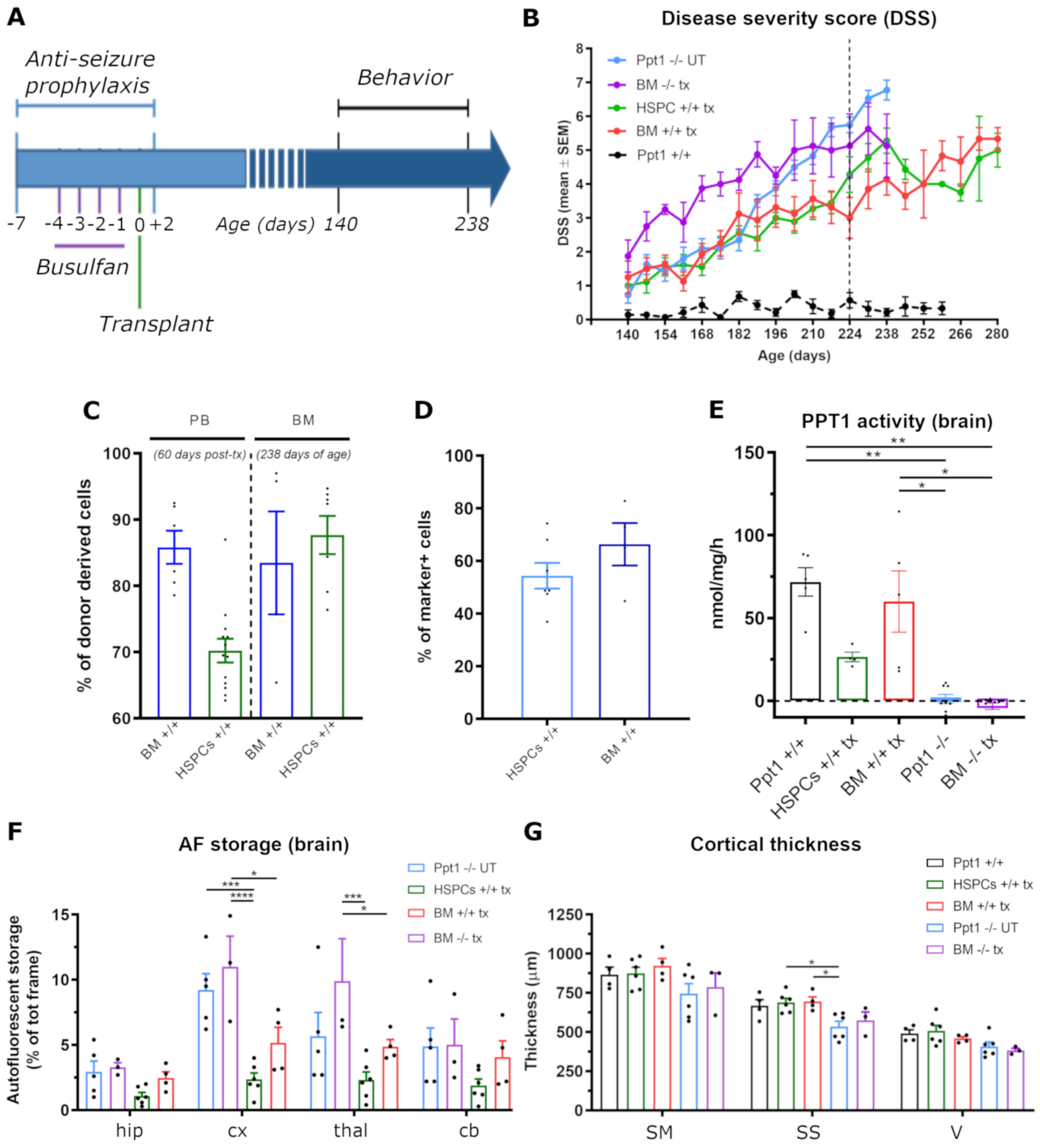

Appendix Fig. S2.

A. Schematic representation of the transplant procedure and animals monitoring post-transplant. **B.** Longitudinal assessment of DSS in HSPCs or BM transplanted mice in comparison with mock transplanted or untreated Ppt1$^{-/-}$ mice. **C.** Assessment of donor-cell engraftment in peripheral blood (PB) and BM of transplanted mice. **D.** Assessment of donor-cell engraftment in the brain of

transplanted mice. **E.** Ppt1 enzymatic activity in brain samples from transplanted mice. * = $p<0.05$; ** = $p<0.01$; Kruskal Wallis followed by Dunn's post-hoc test. **F.** Autofluorescent (AF) storage material in the brain of transplanted mice. * = $p<0.05$; ** = $p<0.01$; *** = $p<0.001$; **** = $p<0.0001$; 2-way ANOVA followed by Tukey's *post-hoc* test. **G.** Assessment of cortical thickness in transplanted mice. * = $p < 0.05$; Kruskal Wallis followed by Dunn's *post-hoc* test (SM); ANOVA followed by Tukey's *post-hoc* test (SS; V).

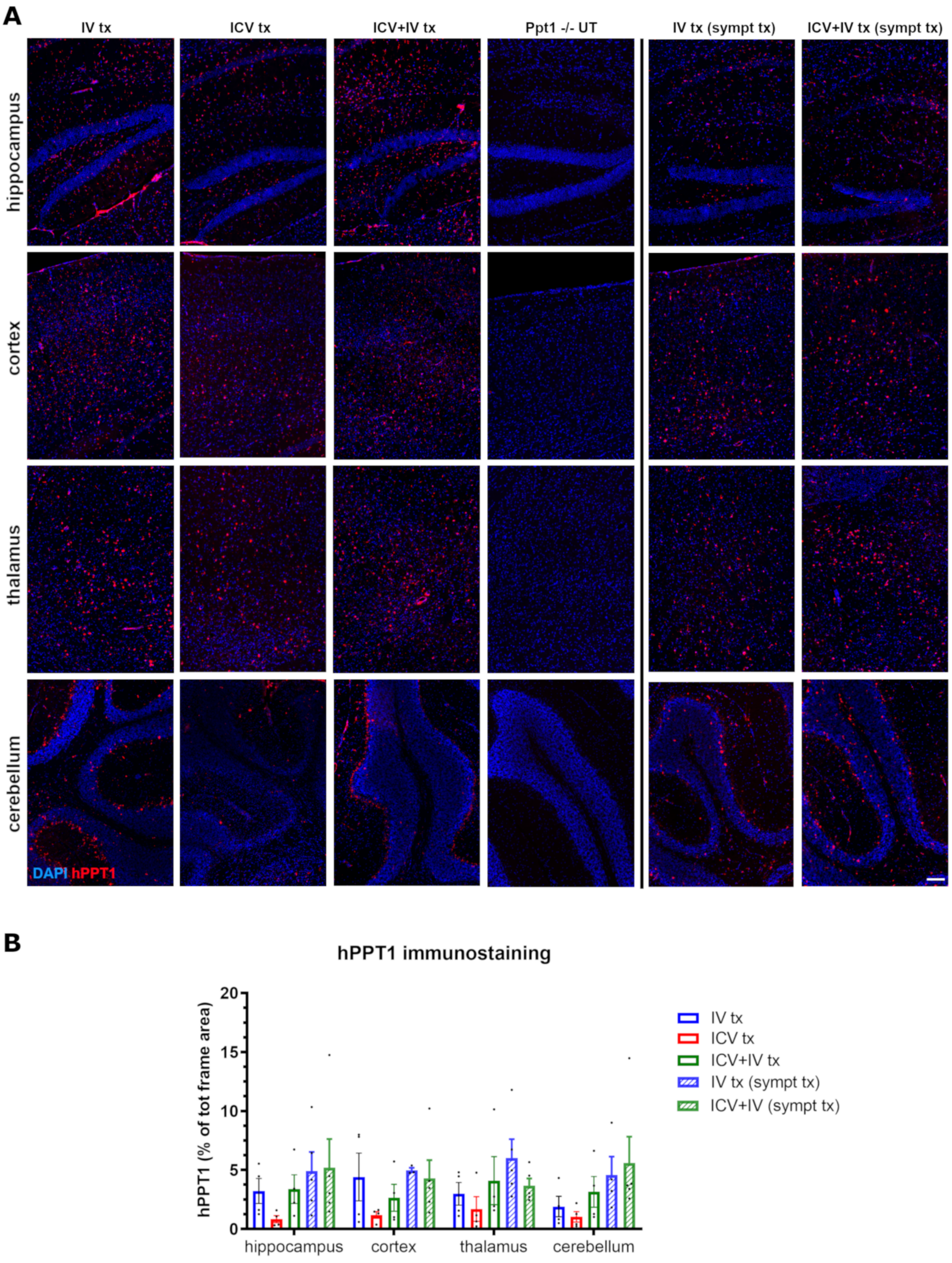

Appendix Fig. S3.

A. Representative fluorescence microscope photomicrographs showing human PPT1 immunoreactivity (hPPT1, red) in different brain regions of $Ppt1^{-/-}$ mice transplanted with hPPT1-LV transduced HSPCs (administered IV, ICV or ICV+IV) at 6-8 weeks of age or at 18-20 weeks of age (sympt tx) analyzed at study termination. $Ppt1^{-/-}$ untreated (UT) mice analyzed at about 250 days, i.e. humane end point, are shown as control of the specificity of hPPT1 staining. DAPI staining (blue) is shown to highlight nuclei. Scale bar = 100 μm. **B.** Quantification of hPPT1 immunostaining in different brain regions of untreated or transplanted mice analyzed at study termination. $p>0.05$; Kruskal Wallis followed by Dunn's post-hoc test.

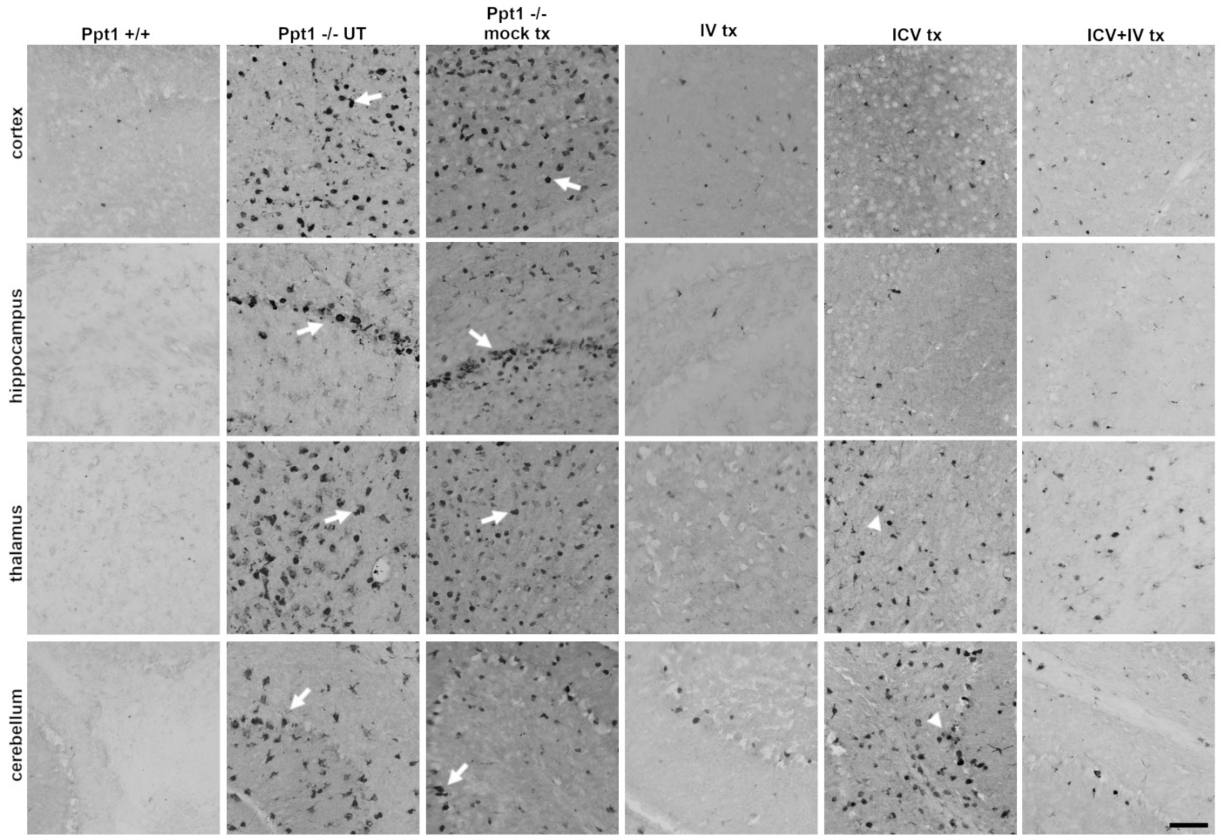

Appendix Fig. S4.

Representative brightfield microscope microphotographs of CD68 immunoreactive DAB staining in the cortex, hippocampus, thalamus and cerebellum, in Ppt1 $^{+/+}$ mice analyzed at 400 days of age, or Ppt1 $^{-/-}$ mice left untreated (UT) or mock transplanted with Ppt1 $^{-/-}$ unmanipulated HSPCs (analyzed at humane end point, ~ 250 days of age); Ppt1 $^{-/-}$ mice transplanted with hPPT1-LV transduced HSPCs administered IV , ICV or ICV+IV , analyzed at study termination, 360-400 days of age. Scale bar = 200 μm.

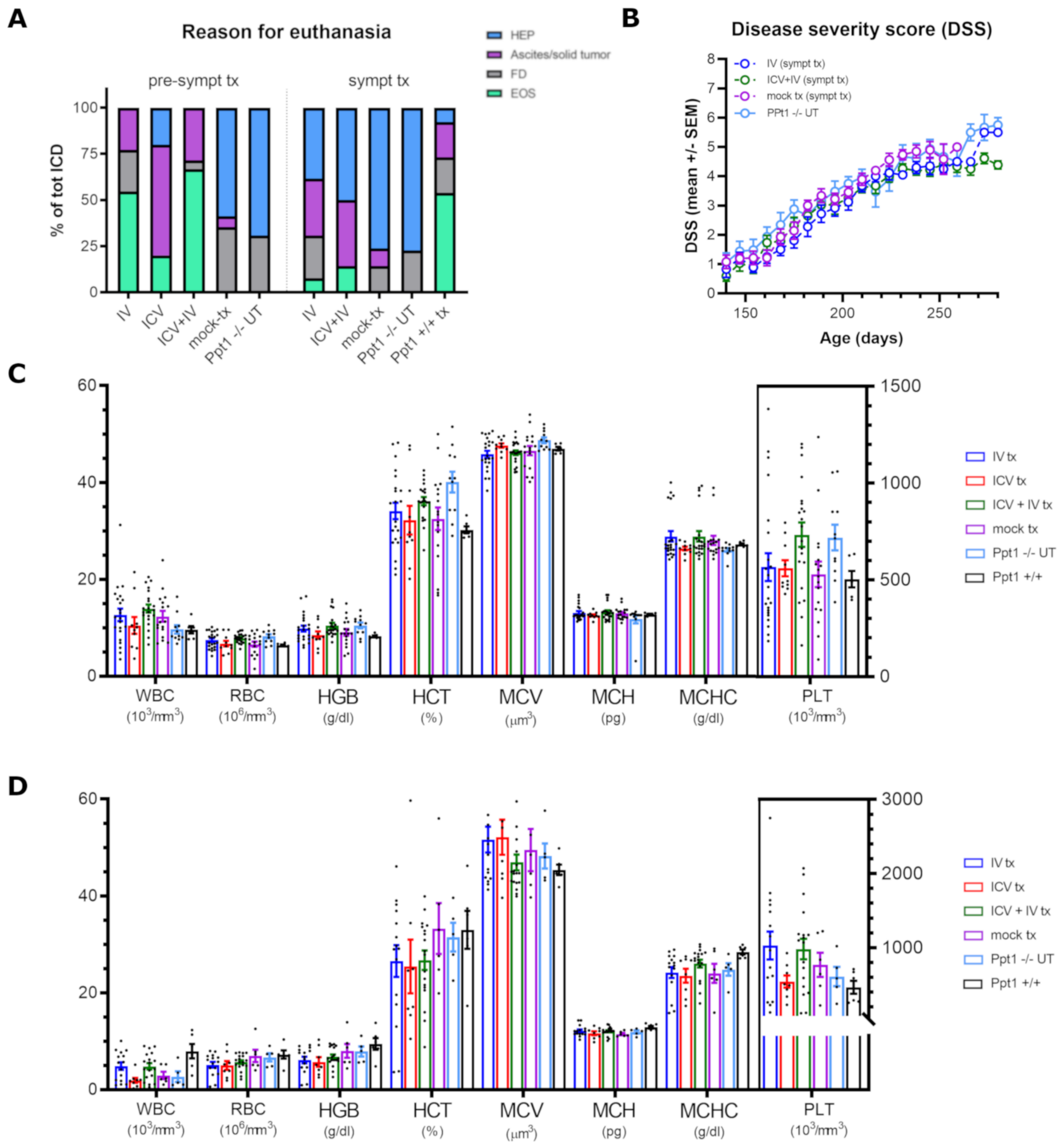

Appendix Fig. S5.

A. Classification of intercurrent deaths (ICD) reported in Ppt1 $^{-/-}$ mice mock transplanted, transplanted with hPPT1-LV transduced HSPCs IV, ICV or ICV+IV, or untreated (UT). HEP = humane end point; FD = found dead; EOS = end of study. **B.** Longitudinal assessment of DSS in HSC-GT and mock transplanted mice treated at the symptomatic stage in comparison with untreated Ppt1$^{-/-}$ mice. See Appendix Table S4 for detailed statistics. **C.** Hemocytometric analysis

of the peripheral blood of transplanted or untreated Ppt1 $^{-/-}$ and Ppt1 $^{+/+}$ mice at 5-6 weeks post-transplant. **D.** Hemocytometric analysis of the peripheral blood of transplanted or untreated Ppt1 $^{-/-}$ and Ppt1 $^{+/+}$ mice at euthanasia. WBC = white blood cells; RBC = red blood cells; HGB = hemoglobin; HCT = hematocrit; MCV = mean corpuscular volume; MCH = mean corpuscular hemoglobin; MCHC = mean corpuscular hemoglobin concentration; PLT = platelets count.

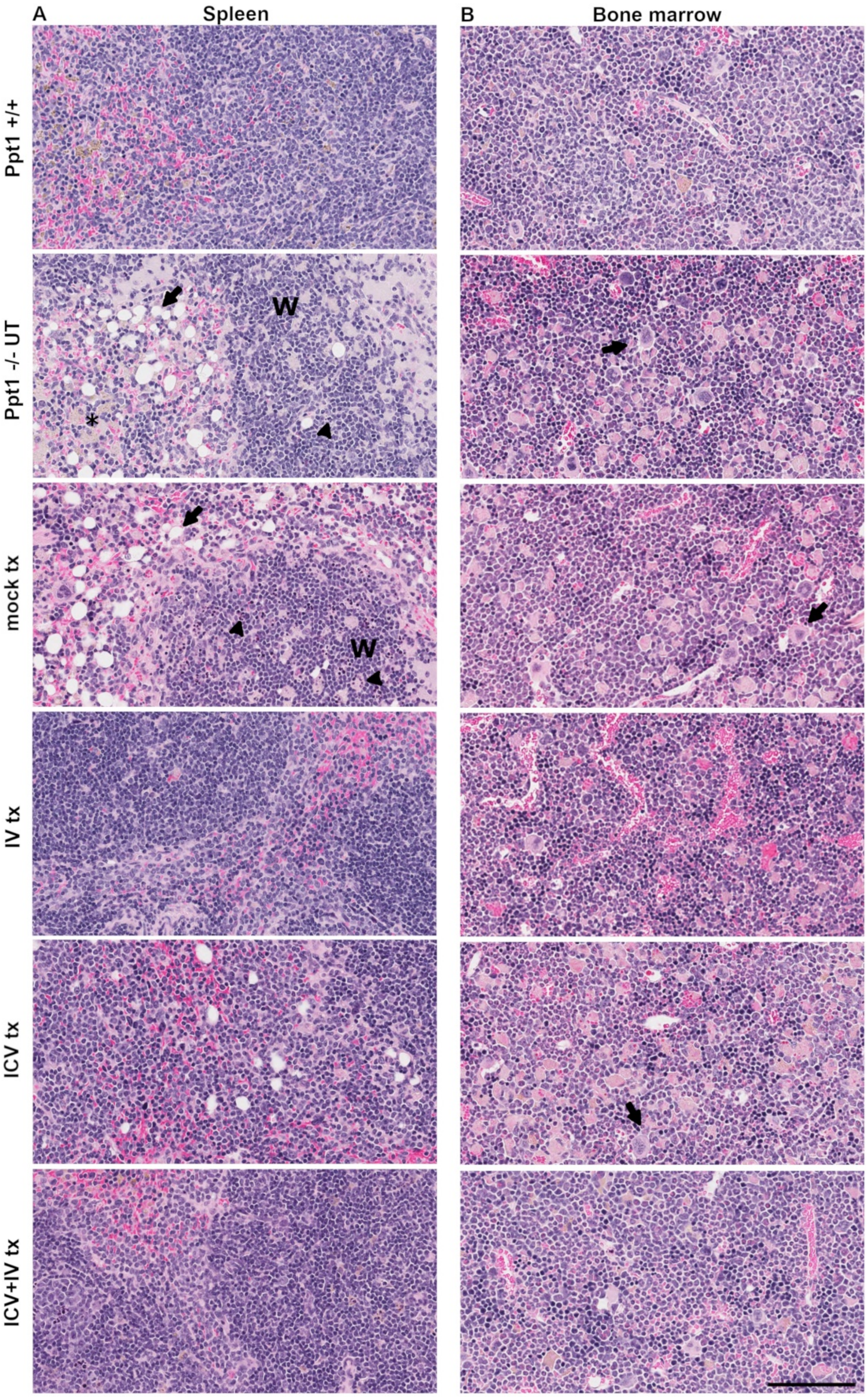

Appendix Fig. S6.

Representative brightfield microscope microphotographs of H&E staining performed on the spleen (**A**) and on the bone marrow (**B**) in Ppt1 $^{+/+}$ mice analyzed at 400 days of age, or Ppt1 $^{-/-}$ mice left untreated (UT) or mock transplanted with Ppt1 $^{-/-}$ unmanipulated HSPCs (analyzed at humane end point, ~ 250 days of age); Ppt1 $^{-/-}$ mice transplanted with hPPT1-LV transduced HSPCs administered IV , ICV or ICV+IV , analyzed at study termination, 360-400 days of age. Scale bar = 100 μm. W = white pulp of the spleen. Arrows in A highlight adipocytes; arrowheads in A highlight apoptotic cells; asterisk highlight the macrophages. Arrows in B highlight macrophage hypertrophy in the bone marrow.

Appendix Table S1. Example of DSS assessment.

Symptoms	Present	Score	TOTAL
Skin wounds	✓	+1	
Seizures (jerks, pop-corn)	✓	+1	
Hind-limbs abduction deficits (close to midline)	✓	+1	
Hind-limbs orizontal displacement			
Hind-limbs clasping	✓	+1	6
Hind-limbs muscle atrophy	✓	+1	
Fore-limbs muscle atrophy			
Fore-limbs clasping			
Limbs stiffness			
Tail-flick	✓	+1	

Appendix Table S2. Log-rank analysis of DSS and disease symptoms manifestation.

Fig. S1D - Log-rank analysis of disease symptoms

	DSS ≥ 2	DSS ≥ 4	DSS ≥ 6	clasping	rotarod	stiffness	survival
**DSS ≥ 2**							
**DSS ≥ 4**	< 0.0001						
**DSS ≥ 6**	< 0.0001	< 0.0001					
**clasping**	ns	< 0.0001	< 0.0001				
**rotarod**	0.0006	ns	< 0.0001	0.0316			
**stiffness**	< 0.0001	< 0.0001	ns	< 0.0001	< 0.0001		
**survival**	< 0.0001	< 0.0001	< 0.0001	< 0.0001	< 0.0001	0.0164	

Appendix Table S3. Statistics for Log-rank analyses among HSC gene therapy groups.

Fig. 2C - Survival

	IV tx	ICV tx	ICV+IV tx	Mock tx	Ppt1 -/- UT
**IV tx**					
**ICV tx**	ns				
**ICV+IV tx**	ns	ns			
**Mock tx**	<0.0001	<0.0001	<0.0001		
**Ppt1 -/- UT**	<0.0001	<0.0001	<0.0001	ns	

Fig. 2E - DSS ≥ 4

	IV tx	ICV tx	ICV+IV tx	Mock tx	Ppt1 -/- UT
**IV tx**					
**ICV tx**	0.0034				
**ICV+IV tx**	ns	<0.0001			
**Mock tx**	0.0009	0.0187	0.005		
**Ppt1 -/- UT**	<0.0001	<0.0001	<0.0001	0.0019	

Fig. 2F - Limb stiffness

	IV tx	ICV tx	ICV+IV tx	Mock tx	Ppt1 -/- UT
**IV tx**					
**ICV tx**	ns				
**ICV+IV tx**	ns	ns			
**Mock tx**	0.0114	0.0107	0.0224		
**Ppt1 -/- UT**	0.0073	0.0193	0.0129	ns	

Fig. 5A - Survival

	IV tx	ICV+IV tx	Mock tx	Ppt1 -/- UT
**IV tx**				
**ICV+IV tx**	0.0066			
**Mock tx**	0.0004	<0.0001		
**Ppt1 -/- UT**	0.0016	<0.0001	ns	

Fig. 5C – DSS ≥ 4

	IV tx	ICV+IV tx	Mock tx	Ppt1 -/- UT
**IV tx**				
**ICV+IV tx**	ns			
**Mock tx**	ns	ns		
**Ppt1 -/- UT**	ns	ns	ns	

Fig. 5D – Limbs stiffness

	IV tx	ICV+IV tx	Mock tx	Ppt1 -/- UT
**IV tx**				
**ICV+IV tx**	ns			
**Mock tx**	0.0048	<0.0001		
**Ppt1 -/- UT**	0.0002	<0.0001	ns	

Appendix Table S4. Statistics for longitudinal analyses among HSC gene therapy groups.

Fig. 2D. Longitudinal assessment of the DSS

A mixed-effect model was applied. The analysis was performed from day 140 to day 350, since more than 50% of values were missing for some groups afterwards (due to ICDs). The comparison was limited only to groups IV tx, ICV tx, ICV+IV tx and Ppt1 +/+ as for the others too many missing values (due to ICDs) were present after day 217.

**Fixed effects**		**p-value**
Time		<0.0001
Group		0.0002
Time x Group		<0.0001
**Tukey's post-hoc test** *(only statistically significant comparisons are shown)*		**p-value**
Day 175	**ICV tx** *vs* Ppt1 +/+ UT	0.0323
Day 182	**IV tx** *vs* Ppt1 +/+ UT	0.0321
	**ICV tx** *vs* Ppt1 +/+ UT	0.0457
Day 189	**ICV tx** *vs* **ICV+IV tx**	0.0209
	**ICV+IV tx** *vs* Ppt1 +/+ UT	0.0284
Day 196	**ICV+IV tx** *vs* Ppt1 +/+ UT	0.0018
Day 203	**ICV+IV tx** *vs* Ppt1 +/+ UT	0.0494
Day 217	**IV tx** *vs* Ppt1 +/+ UT	0.0237
	**ICV+IV tx** *vs* Ppt1 +/+ UT	0.0462
Day 231	**IV tx** *vs* **ICV+IV tx**	0.0179
	**ICV+IV tx** *vs* Ppt1 +/+ UT	0.0063
Day 238	**ICV+IV tx** *vs* Ppt1 +/+ UT	0.0191
Day 245	**ICV+IV tx** *vs* Ppt1 +/+ UT	0.016
Day 252	**ICV tx** *vs* Ppt1 +/+ UT	0.0413
Day 280	**IV tx** *vs* Ppt1 +/+ UT	0.0126
	**ICV tx** *vs* Ppt1 +/+ UT	0.0272
	**ICV+IV tx** *vs* Ppt1 +/+ UT	0.0298
Day 287	**ICV tx** *vs* Ppt1 +/+ UT	0.0307
Day 294	**ICV tx** *vs* Ppt1 +/+ UT	0.0005
Day 308	**IV tx** *vs* Ppt1 +/+ UT	0.0017
	**ICV tx** *vs* Ppt1 +/+ UT	0.0019
	**ICV+IV tx** *vs* Ppt1 +/+ UT	0.0247
Day 315	**IV tx** *vs* Ppt1 +/+ UT	0.0022
	**ICV tx** *vs* Ppt1 +/+ UT	0.0053
	**ICV+IV tx** *vs* Ppt1 +/+ UT	0.006
Day 322	**ICV tx** *vs* Ppt1 +/+ UT	0.0019
	**ICV+IV tx** *vs* Ppt1 +/+ UT	0.0438
Day 336	**IV tx** *vs* Ppt1 +/+ UT	0.001

	**ICV tx** *vs* Ppt1 +/+ UT	0.0011
	**ICV+IV tx** *vs* Ppt1 +/+ UT	0.041
Day 343	**IV tx** *vs* Ppt1 +/+ UT	0.0212
	**ICV tx** *vs* **ICV+IV tx**	0.0023
	**ICV tx** *vs* Ppt1 +/+ UT	<0.0001
Day 350	**IV tx** *vs* **ICV tx**	0.001
	**ICV tx** *vs* **ICV+IV tx**	<0.0001
	**ICV tx** *vs* Ppt1 +/+ UT	0.0004
Day 357	**IV tx** *vs* **ICV+IV tx**	0.0012
	**ICV tx** *vs* **ICV+IV tx**	<0.0001
	**ICV tx** *vs* Ppt1 +/+ UT	0.0053

Fig. 2G. Longitudinal assessment of Rotarod performance

A mixed-effect model was applied. The analysis was performed from day 140 to day 364, since more than 50% of values were missing for some groups afterwards (due to ICDs). The comparison was limited only to groups IV tx, ICV tx, ICV+IV tx and Ppt1 +/+ as for the others too many missing values (due to ICDs) were present after day 217.

**Fixed effects**		**p-value**
Time		<0.0001
Group		0.0039
Time x Group		<0.0001
**Tukey's post-hoc test** *(only statistically significant comparisons are shown)*		**p-value**
Day 252	**IV tx** *vs* **ICV tx**	0.0202
Day 280	**ICV tx** *vs* **ICV+IV tx**	0.0049
	**ICV tx** *vs* Ppt1 +/+ UT	0.0317
Day 308	**IV tx** *vs* **ICV tx**	<0.0001
	**ICV tx** *vs* **ICV+IV tx**	<0.0001
	**ICV tx** *vs* Ppt1 +/+ UT	0.0004
Day 336	**IV tx** *vs* **ICV tx**	0.0008
	**ICV tx** *vs* **ICV+IV tx**	<0.0001
	**ICV tx** *vs* Ppt1 +/+ UT	0.0001
Day 364	**IV tx** *vs* **ICV tx**	<0.0001
	**ICV tx** *vs* **ICV+IV tx**	<0.0001
	**ICV tx** *vs* Ppt1 +/+ UT	<0.0001

Fig. S5B. Longitudinal assessment of DSS in symptomatic transplanted Ppt1 -/- mice.

A mixed-effect model was applied. The analysis was performed from day 140 to day 273, since more than 50% of values were missing for some groups afterwards (due to ICDs). The comparison was limited only to groups IV tx, ICV+IV tx and Ppt1 -/- UT as for the others too many missing values (due to ICDs) were present after day 250.

**Fixed effects**		**p-value**
Time		<0.0001
Group		0.0273
Time x Group		0.1955
**Tukey's post-hoc test** *(only statistically significant comparisons are shown)*		**p-value**
Day 168	**IV sympt-tx** *vs* Ppt1 -/- UT	0.0384
Day 175	**IV sympt-tx** *vs* Ppt1 -/- UT	0.0063
Day 245	**ICV+IV sympt-tx** *vs* Ppt1 -/- UT	0.0197
Day 266	**ICV+IV sympt-tx** *vs* Ppt1 -/- UT	0.0211
Day 273	**ICV+IV sympt-tx** *vs* Ppt1 -/- UT	0.0358

Appendix Table S5. Statistics for the multivariate analyses (Hotelling's T-test).

Fig. 2H – PCA of combined behavioral parameters

	IV tx	ICV tx	ICV+IV tx
IV tx			
ICV tx	0.0811		
ICV+IV tx	0.0442	0.0044	
Ppt1 +/+	<0.0001	0.0044	0.0014

Fig. 4C – PCA of combined histological parameters

	IV tx	ICV tx	ICV+IV tx	Ppt1-/- UT and mock tx
IV tx				
ICV tx	0.0151			
ICV+IV tx	0.1027	0.003		
Ppt1 -/- UT and mock tx	<0.0001	0.0072	<0.0001	
Ppt1 +/+	0.093	0.0015	0.0073	<0.0001

Appendix Table S6. Macroscopic histopathological findings in the abdominal cavity in mice.

Group name	IV tx	ICV tx	ICV+IV tx	Mock tx	Ppt1 -/- UT	Ppt1 +/+ tx	Ppt1 +/+ UT
**Treatment**	$Ppt1^{-/-}$ + HSCPs PPT1-LV (IV)	$Ppt1^{-/-}$ + HSCPs PPT1-LV (ICV)	$Ppt1^{-/-}$ + HSCPs PPT1-LV (IV+ICV)	$Ppt1^{-/-}$ + autologous un-transduced HSCPs IV	$Ppt1^{-/-}$ un-transplanted	$Ppt1^{-/-}$ + autologous un-transduced HSCPs IV	$Ppt1^{+/+}$ un-transplanted
Number of animals in group	13	11	10	11	8	3	3
Mass	2	1	3	0	0	1	1

Appendix Table S7. Microscopic histopathological findings in the abdominal cavity in mice.

Group name	IV tx	ICV tx	ICV+IV tx	Mock tx	Ppt1 -/- UT	Ppt1 +/+ tx	Ppt1 +/+ UT
**Treatment**	$Ppt1^{-/-}$ + HSCPs PPT1-LV (IV)	$Ppt1^{-/-}$ + HSCPs PPT1-LV (ICV)	$Ppt1^{-/-}$ + HSCPs PPT1-LV (IV+ICV)	$Ppt1^{-/-}$ + autologous un-transduced HSCPs IV	$Ppt1^{-/-}$ un-transplanted	$Ppt1^{-/-}$ + autologous un-transduced HSCPs IV	$Ppt1^{+/+}$ un-transplanted
Number of animals in group	13	11	10	11	8	3	3
Sarcoma NOS	3	1	3	0	0	1[a]	0
Atypical Fibroplasia	1	0	0	1	0	1[a]	0
Total	4	1	3	1	0	1	0

[a] sarcoma and atypical fibroplasia occurred in the same animal

Appendix Table S8. Microscopic histopathological findings in the spleen in mice.

Group name	IV tx	ICV tx	ICV+IV tx	Mock tx	Ppt1 -/- UT	Ppt1 +/+ tx	Ppt1 +/+ UT
**Treatment**	$Ppt1^{-/-}$ + HSCPs PPT1-LV (IV)	$Ppt1^{-/-}$ + HSCPs PPT1-LV (ICV)	$Ppt1^{-/-}$ + HSCPs PPT1-LV (IV+ICV)	$Ppt1^{-/-}$ + autologous un-transduced HSCPs IV	$Ppt1^{-/-}$ un-transplanted	$Ppt1^{-/-}$ + autologous un-transduced HSCPs IV	$Ppt1^{+/+}$ un-transplanted
Number of animals in group	13	11	10	11	8	3	3
Decreased Size, Subgross							
Slight	5	6	2	6	5	1	0
Moderate	4	1	3	2	1	0	0
Total	9	7	5	8	6	1	0
Cellularity Decreased, White Pulp							
Slight	4	7	4	4	3	2	0
Moderate	5	1	0	2	2	0	0
Marked	0	0	0	1	1	0	0
Total	9	8	4	7	6	2	0
Apoptosis							
Minimal	1	3	3	4	4	0	2
Slight	1	1	0	1	1	0	0
Moderate	0	1	0	1	0	0	0
Total	2	5	3	6	5	0	2
Aggregates, Macrophage							
Minimal	0	2	0	1	0	0	0
Slight	2	4	0	5	6	0	0
Moderate	0	0	0	2	1	0	0
Total	2	6	0	8	7	0	0
Adipocytes							
Minimal	1	3	0	0	1	0	0
Slight	1	1	0	5	4	0	0
Moderate	0	1	0	1	1	0	0
Total	2	5	0	6	6	0	0

Appendix Table S9. Microscopic histopathological findings in the bone marrow in mice.

Group name	IV tx	ICV tx	ICV+IV tx	Mock tx	Ppt1 -/- UT	Ppt1 +/+ tx	Ppt1 +/+ UT
**Treatment**	**$Ppt1^{-/-}$ + HSCPs PPT1-LV (IV)**	**$Ppt1^{-/-}$ + HSCPs PPT1-LV (ICV)**	**$Ppt1^{-/-}$ + HSCPs PPT1-LV (IV+ICV)**	**$Ppt1^{-/-}$ + autologous un-transduced HSCPs IV**	**$Ppt1^{-/-}$ un-transplanted**	**$Ppt1^{-/-}$ + autologous un-transduced HSCPs IV**	**$Ppt1^{+/+}$ un-transplanted**
Number of animals in group	13	11	10	11	8	3	3
Cellularity, Increased, Macrophage							
Minimal	0	0	1	0	0	0	0
Slight	6	3	3	3	2	0	0
Moderate	0	4	0	6	5	0	0
Total	6	7	4	9	7	0	0
