# Supplementary material for: An innovative hematopoietic stem cell gene therapy approach benefits CLN1 disease in the mouse model
Source: EMBO Mol Med. 2023 Mar 6;15(4):e15968. doi: 10.15252/emmm.202215968 (PMC10086581; doi:10.15252/emmm.202215968)
Supplement: Supplementary file 7 [file EMMM-15-e15968-s007.pdf]

EMBO Molecular Medicine

An innovative hematopoietic stem cell gene therapy approach benefits CLN1 disease in the mouse model

Marco Peviani, Sabyasachi Das, Janki Patel, Odella Jno-Charles, Rajesh Kumar, Ana Zguro, Tyler Mathews, Paolo Cabras, Rita Milazzo, Eleonora Cavalca, Valentina Poletti, and Alessandra Biffi
DOI: 10.15252/emmm.202215968

Corresponding author: Alessandra Biffi (alessandra.biffi@unipd.it)

Review Timeline:

Submission Date:	4th Mar 22
Editorial Decision:	21st Apr 22
Revision Received:	15th Oct 22
Editorial Decision:	18th Nov 22
Revision Received:	25th Jan 23
Accepted:	9th Feb 23

Editor: Zeljko Durdevic

Transaction Report:

(Note: With the exception of the correction of typographical or spelling errors that could be a source of ambiguity, letters and reports are not edited. Depending on transfer agreements, referee reports obtained elsewhere may or may not be included in this compilation. Referee reports are anonymous unless the Referee chooses to sign their reports.)

21st Apr 2022

Dear Prof. Biffi,

Thank you for the submission of your manuscript to EMBO Molecular Medicine, and please accept my apologies for the delay in getting back to you. We have now received feedback from two of the three reviewers who agreed to evaluate your manuscript. Given that referee #3 will unfortunately not be able to return his/her report in a timely manner, and that both referees #1 and #2 gave similar recommendation, we prefer to make a decision now in order to avoid further delay in the process. Should referee #3 provide a report, we will send it to you, with the understanding that we will not ask for an additional revision. As you will see from the reports below, the referees acknowledge the interest of the study but also raise serious and partially overlapping concerns that should be addressed in a major revision.

Further consideration of a revision that addresses reviewers' concerns in full will entail a second round of review. EMBO Molecular Medicine encourages a single round of revision only and therefore, acceptance or rejection of the manuscript will depend on the completeness of your responses included in the next, final version of the manuscript. For this reason, and to save you from any frustrations in the end, I would strongly advise against returning an incomplete revision.

We would welcome the submission of a revised version within three months for further consideration. Please let us know if you require longer to complete the revision.

I look forward to receiving your revised manuscript.

Yours sincerely,

Zeljko Durdevic

Zeljko Durdevic
Editor
EMBO Molecular Medicine

***** Reviewer's comments *****

Referee #1 (Comments on Novelty/Model System for Author):

it is unavoidable to use an animal model such as mouse

Referee #1 (Remarks for Author):

This work describes the development of lentiviral gene therapy for CLN1, and it adds and additional aspect to better treat the CNS using a combination between IV and ICV administration. This is an interesting study, although it remains to be seen how safe the additional ICV treatment will be. Have the authors performed safety studies for the CNS and SC, such as genotoxicity assays? In any case, it will be important to discuss these aspects better in the manuscript to highlight the need for such studies before clinical implementation can be considered.

The VCN in brain after IV administration is surpringly high, around 0.75 copies/genome. This contrasts with reports in the literature, in which this value is usually an order of magnitude smaller. It might be related to the relatively high VCN in PB, around 9. This raises doubts to the clinical translation of the results obtained, as lower VBC are usually applied. This is an important aspect of the work, and the authors should expand on this. Have they tried lower lentiviral dosage? Why are VCNs so high in this study?

Other comments:

1) In figure legends for figure 2B, 3A and 5B authors refer to graphs of VCN as graphs for chimerism
2) In figure 5 legend, "A" and "B" should be in bold
3) The authors should justify the use of showing the same data in 2 different graphs (Figure 1F and S2F; Figure 2E and 1E)
4) Authors should discuss about the clinical relevance of the relatively high VCN in relation to genotoxic events
5) Authors should plot WT mice in survival curves
6) As for DSS assessment, authors state "weekly DSS assessment and monthly rotarod testing demonstrated that the majority of the gene therapy treated animals were protected from the development of severe neurological symptoms. Indeed, while UT and mock-transplanted animals experienced a progressive neurological deterioration after the age of 220 days, with half of them in disease stage 2 (DSS {greater than or equal to} 4) at 220-240 days of age (Fig. 2D-E), the majority of the transplanted mice showed no clinical onset of the disease (DSS {less than or equal to} 2) for the whole duration of the observation (Fig. 2G)."

DSS neurological symptoms assessment is limited to abnormal limb displacement when the animal is raised, paws clasping behavior and motor coordination in rotarod test. DSS does not include assessment of learning or memory ability. Authors should comment on the limitations of DSS assessment.

Authors state in the discussion that ICV administration is likely to treat CNS manifestation and not extra CNS manifestation. This disagrees with performance of ICV treated mice in DSS curves. In fact, ICV treated mice degenerate over time on DSS assessment. Is this biased by the fact that DSS assessment is not specific for neurological symptoms or simply the hypothesis that ICV treatment only treats CNS manifestations does not stand? Authors should adjust discussion and comment on this.

7) Authors state: "Multivariate analysis of variance and Hotelling's T-test confirmed a significant difference between animals transplanted IV+ICV and the animals that received the cells only IV ($p<0.05$) or ICV ($p<0.01$), suggesting that the combinatorial transplant strategy could achieve greater (and more homogeneous across subjects) clinical benefit than the individual approaches". Authors should also report statistics vs controls

8) Figure 3A: authors should comment on discrepancies between activity and VCN: after ICV treatment, VCN is lower in brain than in SP. On the other hand, activity is higher in brain than in SC in ICV treated animals. In addition, activity in brain is comparable between IV tx animals and ICV tx animals, while VCN is higher for IV tx animals compared to ICV tx animals. Since the main mechanism of correction proposed by authors is engraftment in CNS, such a discrepancy should be addressed in the discussion section

9) Figure 3 C authors should indicate markers/probes used as inserts to facilitate interpretation.

10) Authors should report number of animals used in each experimental group in figure legends

11) Figure S4C and S4D authors evaluate peripheral blood composition. Was this analysis performed using a normal cell counter of with FACS analysis? Authors state that there is no clonal expansion. Did authors check specific lymphocyte populations? Clonal expansion analysis requires a more extensive analysis of lymphocytes population.

12) Authors mention but do not show histopathological analysis of hematopoietic organs. Authors should show this data

13) In the discussion authors state: Indeed, at study termination we detected lower chimerism and Ppt1 activity in the brain and spinal cord of ICV-transplanted mice as compared to the animals that received HSC gene therapy IV.

This is not shown: authors showed lower VCN (not chimerism) in ICV vs IV treated mice and lower activity in spinal cord, not in brain (fig 3 A, B). Authors should adjust this part

14) Authors should show AF analysis in brain of mice treated at later stage of disease progression.

Referee #2 (Remarks for Author):

Pevani and colleagues investigated hematopoietic stem cell (HSC) therapy in a mouse model of neuronal ceroid lipofuscinosis 1 (CLN1), a progressive and devastating disease that is currently lacking treatment. The main findings of the study are the proof-of-efficacy of transplantation of wild-type HSC in disease mitigation and transplantation of genetically corrected HSC by peripheral infusion (IV) alone or in combination with intracerebroventricular (ICV) injections. The authors claimed an improved efficacy of HSC lentiviral vector mediated gene correction over the bone marrow transplantation. However, the two approaches are not compared head-to-head and it is difficult to draw such conclusion from the presented data.
In the pre-symptomatic mice, the data do not clear support that the outcomes of the ICV+IV group is better in terms of efficacy compared to the ICV or IV groups alone. Only by the PCA analysis, the ICV+IV group performs better than the other groups. Moreover, the statement about the faster efficacy of the ICV+IV group is not supported by the data. Although survival improved in the symptomatic mice, no significant differences were detected on enzyme activity, DSS and limb stiffness. Given the challenges of this route of administration and the potential concerns (e.g., brain cancer), the data should clearly support the superiority of the ICV+IV route over the standard IV route, at least in pre-symptomatic patients.
The authors should also discuss whether there are concerns of ICV injections related to brain cancer and the possibility of combining injection of viral vectors (e.g. AAV) in the brain combined with ex vivo HSC gene therapy as alternative option.
Although the authors measured PPT1 activity in the brain, there were no other measures on cell engraftment in the brain. For example, staining for the PPT1 in various areas of the brain would have been informative.
The authors argued that storage in peripheral tissues might occur in CLN1. This hypothesis could be easily addressed by AF of the heart, at least.

Major remarks

1. The DSS score figure 1 should have included a wt control group.
2. Fig. 1F: lines with the statistical comparisons are not aligned to the bars.
3. Fig. 1H: the rescue of visual cortex thickness was not found as there is no statistically significant difference between treated and untreated mice.
4. Fig. 2: the labeling of the y axis is switched: the VCN should have the logarithmic scale and the enzyme activity the non-logarithmic scale.
5. Fig. 2: on survival, DSS, limb stiffness and rotarod, there is no significant difference between IV and IV+ICV. Therefore, I do not understand how the multivariate analysis can show a statistical difference and even if that is the case, I wonder about the clinical relevance of this multivariate analysis.
6. Fig. 3A: by VCN and enzyme activity, authors are not evaluating cell engraftment. This will be evaluated by immunochemistry looking at the number of corrected cells in the brain.
7. Fig. 3C: AF signal in the 'mock tx' group appears to be reduced across all brain areas compared to the untreated group. The quantification shown in fig. 3D does not show a reduction, but I wonder whether representative images were selected.
8. AF immunofluorescence should be performed also in the mice receiving ICV + IV treatment after the onset of the symptoms.
9. The authors reported that transplanted mice evaluated for full necropsy. Did they look specifically for brain tumors? Were brain tumors ruled out and how?

Minor issues
1. Chromosomal location of PPT1 gene is not needed (page 3).
2. The English requires attention. For example:
a. Page 4: '...HSC gene therapy my address this goal' rather than 'these indications'.
b. Page 4: 'proof of concept' rather than 'proof of applicability'.
c. Page 5: '6-8 week-old' rather than '6-8 weeks-old).
d. Page 5 'administered with busulfan' rather than 'exposed to a busulfan-based'.
e. What is 'dose proof'?
3. Please do not use 'PBC' acronym for 'poor body condition' as 'PBC' is typically used for peripheral blood cells'.
4. Check that figures are numbered in the order they appear in the text: Fig. 5C is cited before 5B; Fig. S3 is cited for the first time on page 11 (after fig. S4).

***** Reviewer's comments *****

Referee #1 (Comments on Novelty/Model System for Author):
it is unavoidable to use an animal model such as mouse

Referee #1 (Remarks for Author):
This work describes the development of lentiviral gene therapy for CLN1, and it adds and additional aspect to better treat the CNS using a combination between IV and ICV administration. This is an interesting study, although it remains to be seen how safe the additional ICV treatment will be. Have the authors performed safety studies for the CNS and SC, such as genotoxicity assays? In any case, it will be important to discuss these aspects better in the manuscript to highlight the need for such studies before clinical implementation can be considered.

> ***Answer***: We performed an extensive toxicological evaluation that was run in parallel to the efficacy assessment. The evaluation included, after formal dose proof, a regular (every working day) observation for clinical signs/vitality, early detection of intercurrent deaths, hematology evaluation at dose proof and natural death/sacrifice, full necropsy at natural death/sacrifice, histopathological investigations on multiple tissues by expert pathologists (outsourced) and in particular on the CNS, where evaluation of hallmarks of neurodegeneration and neuroinflammation was also performed. These analyses excluded potential neurotoxicity caused by the cell administration in all study animals, including those in which the transduced HSPCs were administered ICV. As far as genotoxicity is concerned, we would respectfully note that it depends on the effects of lentiviral vector integration on the DNA of transduced HSPCs and it is not expected to be influenced by the route of cell administration. As also described in the answer to question nr. 4 below, previous preclinical investigations as well as clinical data thus far have not shown genotoxic effects induced by HSPCs transduction with the lentiviral vector construct employed in this study that is nowadays considered a valuable and safe tool for HSC gene therapy clinical development and use.

The VCN in brain after IV administration is surpringly high, around 0.75 copies/genome. This contrasts with reports in the literature, in which this value is usually an order of magnitude smaller. It might be related to the relatively high VCN in PB, around 9. This raises doubts to the clinical translation of the results obtained, as lower VBC are usually applied. This is an important aspect of the work, and the authors should expand on this. Have they tried lower lentiviral dosage? Why are VCNs so high in this study?

> ***Answer:*** As described in the material and methods section, HSPCs were transduced with lentiviral vectors at a MOI of 100 according to a standard protocol which has been used for this kind of studies by others and us since 2005. This protocol results in efficient murine HSPC transduction with resulting in vitro and in vivo VCNs ranging between 5 and 10. These values were for instance obtained in the proof of concept (POC) studies that led to

the clinical testing of HSPC gene therapy in Metachromatic Leukodystrophy (MLD) and Mucopolysaccharidosis type I (MPS I). The results obtained in the murine models well translated into the clinics in the two latter indications, even if on average the human drug products (patient's HSPCs transduced with clinical grade LVs) had on average a slightly lower VCN as compared to their murine equivalents employed in the POC studies. Among the reasons for this, we would comment on the human PGK promoter, that drives PPT1 expression in the LV employed in this manuscript as well as arylsulfatase A and alpha-iduronidase expression in the MLD and MPS I settings. Actually, this promoter is known for an expected higher activity in human HSPCs as compared to their murine equivalents, thus allowing to predict that results obtained with a VCN of 5-10 in CLN1 mice could translate in similar outcomes when employing human HSPCs bearing on average a lower VCN, i.e. 2-6 copies/cell.

Other comments:

1) In figure legends for figure 2B, 3A and 5B authors refer to graphs of VCN as graphs for chimerism

Answer: Based on Reviewer's note, we have changed the reference in figure legends replacing "donor cell chimerism" with "number of vector copies" to better reflect the actual data shown in the figure.

2) In figure 5 legend, "A" and "B" should be in bold

Answer: We have adjusted the figure legend according to Reviewer's suggestion.

3) The authors should justify the use of showing the same data in 2 different graphs (Figure 1F and S2F; Figure 2E and 1E)

Answer: The experimental groups, as highlighted in the materials and methods section and in the main text, comprised Ppt1$^{-/-}$ untreated animals, Ppt1$^{-/-}$ busulfan conditioned mice transplanted with BM from Ppt1$^{+/+}$ mice or with purified hematopoietic stem and progenitor (lineage negative) cells from Ppt1$^{+/+}$ mice or with BM retrieved from Ppt1$^{-/-}$ mice. The behavioral analysis (shown in Fig. S2B) and the biochemical and histological analyses (shown in Fig. S2E and Fig. S2F, G, respectively) did not highlight any statistical difference between the group of mice transplanted with Ppt1$^{+/+}$ BM or with Ppt1$^{+/+}$ HSPCs. Based on these results, we decided to combine in one single group (named "Ppt1$^{+/+}$ BM or HSPCs") the behavioral, histological and biochemical data obtained from the two groups of transplanted mice. The combined data were shown in the main figure (**Fig.1**), but, for clarity, we decided to show the data from the single groups (either Ppt1$^{+/+}$ BM or HSPCs) in the supplementary Figures. This explanation has been added in the main text with the following sentence: "*Disease progression was superimposable between Ppt1$^{-/-}$ mice transplanted with Lin^{-} or BM WT cells (Fig. S2B); for this reason, the two groups were combined to highlight in Fig.1 the overall clinical benefit exerted by the transplanted Ppt1$^{+/+}$ hematopoietic cells, in comparison with untreated and mock-transplanted Ppt1$^{-/-}$ mice*".

4) Authors should discuss about the clinical relevance of the relatively high VCN in relation to genotoxic events

Answer: As discussed above and described in the material and methods section, HSPCs were transduced with lentiviral vectors at a MOI of 100 according to a standard protocol which has been used for this kind of studies by others and us since 2005. This protocol results in efficient murine HSPC transduction with resulting in vitro and in vivo VCNs ranging between 5 and 10. These values were for instance obtained in the POC studies that led to the clinical testing of HSPC gene therapy in MLD and MPS I. The results obtained in the murine models well translated into the clinics in the two latter indications, even if on average the human drug products (patient's HSPCs transduced with clinical grade LVs) had on average a slightly lower VCN as compared to their murine equivalents employed in the POC studies. Among the reasons for this, we would comment on the human PGK promoter, that drives PPT1 expression in the LV employed in this manuscript as well as arylsulfatase A and alpha-iduronidase expression in the MLD and MPS I settings. Actually, this promoter is known for an expected higher activity in human HSPCs as compared to their murine equivalents, thus allowing to predict that results obtained with a VCN of 5-10 in CLN1 mice could translate in similar outcomes when employing human HSPCs bearing on average a lower VCN, i.e. 2-6 copies/cell. The safety of this transduction conditions has been extensively demonstrated in mice and patients, with a follow up data reaches almost 12 years post-transplant for the first MLD children. In both the preclinical and clinical setting no adverse genotoxic events, nor shewing of the integration profile of the vector nor abnormal clonal dominance events have been observed, testifying that efficient transduction with this LV backbone carrying lysosomal enzyme cDNA driven by the human PGK promoter is not associated to genotoxic effects, as far as the current observation can tell. Of note, genotoxicity is mostly dependent on the intrinsic features (regulatory elements and integration site preference in the genome) of the LV construct, rather than the actual vector load/VCN.
Of note, despite in this manuscript we focused our attention on the efficacy and overall safety of the procedure (with documentation of general toxicity, in terms of general hematology, pathology and tumorigenesis in the mouse model), no abnormal findings that could suggest genotoxic events have been noticed with an observation of over 10 months post-transplant More specific genotoxicity studies will be completed on human HSPCs on the path to clinical translation, but they fall beyond the scope of this study. For this reason, samples necessary to investigate vector integration sites have been collected during the study but, as described in the text of the manuscript, they will be analyzed in a future follow-up study.

5) Authors should plot WT mice in survival curves

Answer: We added the WT ($Ppt1^{+/+}$) mice in the plot of the survival curves in Fig.1B, in Fig. 2C and in Fig. 5A (as suggested by the Reviewer).

6) As for DSS assessment, authors state "weekly DSS assessment and monthly

rotarod testing demonstrated that the majority of the gene therapy treated animals were protected from the development of severe neurological symptoms. Indeed, while UT and mock-transplanted animals experienced a progressive neurological deterioration after the age of 220 days, with half of them in disease stage 2 (DSS {greater than or equal to} 4) at 220-240 days of age (Fig. 2D-E), the majority of the transplanted mice showed no clinical onset of the disease (DSS {less than or equal to} 2) for the whole duration of the observation (Fig. 2G)."

DSS neurological symptoms assessment is limited to abnormal limb displacement when the animal is raised, paws clasping behavior and motor coordination in rotarod test. DSS does not include assessment of learning or memory ability. Authors should comment on the limitations of DSS assessment.

> ***Answer:*** Following reviewer's suggestion, we have taken in consideration the limitations of the DSS (as far as the assessment of learning and memory is concerned) and we have accordingly modified the description of the results by removing any reference to "neurological symptoms" and describing, instead, the specific symptoms measured by the DSS.

Authors state in the discussion that ICV administration is likely to treat CNS manifestation and not extra CNS manifestation. This disagrees with performance of ICV treated mice in DSS curves. In fact, ICV treated mice degenerate over time on DSS assessment. Is this biased by the fact that DSS assessment is not specific for neurological symptoms or simply the hypothesis that ICV treatment only treats CNS manifestations does not stand? Authors should adjust discussion and comment on this.

> ***Answer:*** Based on our behavioral observations, shown in figure 2C through G, ICV-transplanted animals start displaying a progressive worsening of symptoms after 250 days of age. Considering that the DSS (as we highlighted in the previous answer) does not pick neurological symptoms specifically, the overall gradual decrease of performance registered for ICV-transplanted animals through the DSS and the rotarod might highlight also the involvement of peripheral nerves and/or muscle degeneration. Thus, we believe that our hypothesis that ICV-transplanted cells might treat only CNS-specific manifestations could stand. However, as mentioned in the discussion, we also propose that ICV-transplanted cells could eventually be partially exhausted in the long-term or that their distribution could not be sufficient for providing rescue of disease pathology in some CNS regions. We thus believe that ICV treatment benefits to some extent CNS pathology, but these effects are not sufficient for an entire rescue and obviously do not include the extra-CNS tissues. We revised the discussion to render these concepts clearer to the reader.

7) Authors state: "Multivariate analysis of variance and Hotelling's T-test confirmed a significant difference between animals transplanted IV+ICV and the animals that received the cells only IV ($p<0.05$) or ICV ($p<0.01$), suggesting that the combinatorial transplant strategy could achieve greater (and more homogeneous across subjects) clinical benefit than the individual approaches". Authors should also report statistics vs controls

> ***Answer:*** Following the Reviewer's suggestion, we added the results of

Hotelling's t-test vs WT in the supplementary table 4 of the revised manuscript.

8) Figure 3A: authors should comment on discrepancies between activity and VCN: after ICV treatment, VCN is lower in brain than in SP. On the other hand, activity is higher in brain than in SC in ICV treated animals. In addition, activity in brain is comparable between IV tx animals and ICV tx animals, while VCN is higher for IV tx animals compared to ICV tx animals. Since the main mechanism of correction proposed by authors is engraftment in CNS, such a discrepancy should be addressed in the discussion section

Answer: To address this issue, we plotted the VCN versus the corresponding PPT1 activity retrieved in brain or in the spinal cord for IV- or ICV-transplanted mice. As shown in the figure, while there was no correlation between VCN and PPT1 in the brain of IV-transplanted mice (A), we found a direct correlation between VCN and PPT1 in the brain of ICV-transplanted mice (C). On the other hand, we retrieved the opposite result in the spinal cord (B and D, respectively). We hypothesized that the route of transplantation might determine uneven distribution of the PPT1-expressing cells in specific CNS regions, leading to a discrepancy between the amount of PPT1 enzymatic activity and the VCN retrieved in the brain and in the spinal cord. On the other hand, the ICV+IV combinatorial strategy could achieve a more widespread distribution of cells (and accordingly of PPT1) in different brain and spinal cord regions, overcoming the limitations of the single administration strategies (see E and F).

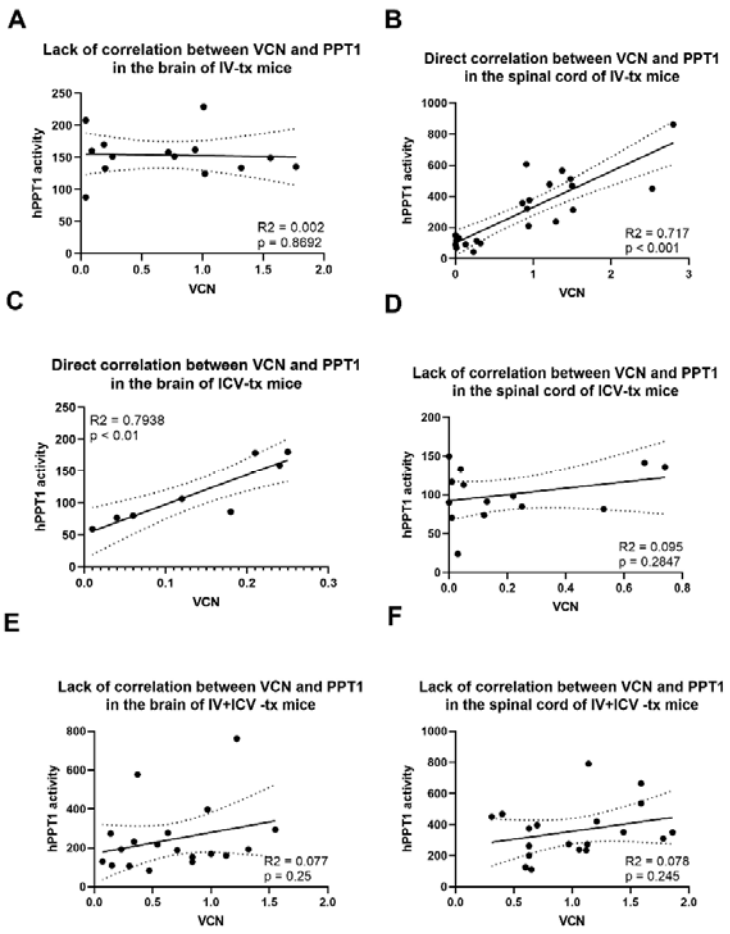

9) Figure 3 C authors should indicate markers/probes used as inserts to facilitate interpretation.

Answer: Following the Reviewer's suggestion. We added the indication of markers used as inserts in the main figure which is now numbered Fig.3E in the revised version of the manuscript.

10) Authors should report number of animals used in each experimental group in figure legends

Answer: To make the figures and legends more understandable, we decided to add the number of animals used in each experimental group in the material and methods section. In the figures, whenever histograms are shown, we highlight the number of animals/group with dots (one per each animal) on the graph.

11) Figure S4C and S4D authors evaluate peripheral blood composition. Was this analysis performed using a normal cell counter of with FACS analysis? Authors state that there is no clonal expansion. Did authors check specific lymphocyte populations? Clonal expansion analysis requires a more extensive analysis of lymphocytes population.

Answer: Peripheral blood composition was performed through an hemocytometric analysis measuring WBC and subpopulation, RBC, Hb etc…., followed by a FACS analysis studying the CD45+ population with antibodies recognizing B220, CD3 and CD11b for B lymphocytes, T lymphocytes and myeloid cells, respectively. Since no expansion of individual populations beyond normal values (namely values observed in age matched WT controls) was observed, the data were not shown, but are available if helpful for the Reviewer or to be included in the Supplementary material.

12) Authors mention, but do not show histopathological analysis of hematopoietic organs. Authors should show this data

Answer: According to the Reviewer's suggestion, we have added a new supplementary figure (Fig. S6) where representative H&E staining of spleen and BM are shown. The observations described in the text are highlighted by arrows in the corresponding Fig.S5.

13) In the discussion authors state: Indeed, at study termination we detected lower chimerism and Ppt1 activity in the brain and spinal cord of ICV-transplanted mice as compared to the animals that received HSC gene therapy IV.

This is not shown: authors showed lower VCN (not chimerism) in ICV vs IV treated mice and lower activity in spinal cord, not in brain (fig 3 A, B). Authors should adjust this part

Answer: According to the Reviewer's suggestion, we have adjusted the discussion as follows: "*Indeed, at study termination <u>we detected lower VCN in the brain and spinal cord of ICV-transplanted mice</u> as compared to the animals that received HSC gene therapy IV……… Moreover, the biodistribution of ICV-transplanted HSPCs in the CLN1 disease mouse brain could be inefficient or uneven in some regions, which could explain*

the striking accumulation of AF storage in the cerebellum and in the thalamus. Similarly, limited engraftment in the spinal cord (paralleled by the lower Ppt1 activity retrieved in this compartment for the ICV transplanted group) could be of relevance, as spinal cord pathology is considered of increasing importance in the Ppt1-/- mouse."

14) Authors should show AF analysis in brain of mice treated at later stage of disease progression.

Answer: AF analysis has been performed on the brain of mice transplanted at the symptomatic stage. The results are now shown in Fig. 5 of the revised manuscript.

Referee #2 (Remarks for Author):

Pevani and colleagues investigated hematopoietic stem cell (HSC) therapy in a mouse model of neuronal ceroid lipofuscinosis 1 (CLN1), a progressive and devastating disease that is currently lacking treatment. The main findings of the study are the proof-of-efficacy of transplantation of wild-type HSC in disease mitigation and transplantation of genetically corrected HSC by peripheral infusion (IV) alone or in combination with intracerebroventricular (ICV) injections. The authors claimed an improved efficacy of HSC lentiviral vector mediated gene correction over the bone marrow transplantation. However, the two approaches are not compared head-to-head and it is difficult to draw such conclusion from the presented data.
In the pre-symptomatic mice, the data do not clear support that the outcomes of the ICV+IV group is better in terms of efficacy compared to the ICV or IV groups alone. Only by the PCA analysis, the ICV+IV group performs better than the other groups. Moreover, the statement about the faster efficacy of the ICV+IV group is not supported by the data. Although survival improved in the symptomatic mice, no significant differences were detected on enzyme activity, DSS and limb stiffness. Given the challenges of this route of administration and the potential concerns (e.g., brain cancer), the data should clearly support the superiority of the ICV+IV route over the standard IV route, at least in pre-symptomatic patients.
The authors should also discuss whether there are concerns of ICV injections related to brain cancer and the possibility of combining injection of viral vectors (e.g. AAV) in the brain combined with ex vivo HSC gene therapy as alternative option.

> ***Answer:*** We understand the concerns of the Reviewer and appreciate his/her thoughtful revision of the manuscript. The role of combined IV and ICV delivery is different in pre- vs symptomatic treated animals. In the former setting, the ICV+IV group was very homogenous in its performance at all the different testing and displayed histological and behavioral features more similar to WT animals than the animals treated by the other modalities; this resulted in a statistically superior therapeutic potential as compared to the IV- and ICV-only approaches at multivariate analyses. In the latter (symptomatic) setting, ICV+IV treated mice significantly outlived not only the UT and mock-transplanted control mice, but also the IV treated cohort, and displayed hindlimb stiffness (which represents the most evident symptom displayed by Ppt1 -/- animals when they reach the advanced stage of the pathology) only in 20% of cases, supporting the increased therapeutic potential of the approach, particularly relevant in this challenging setting. Similarly, the ICV+IV treated mice showed a higher VCN, and correspondingly higher enzymatic activity, in the brain and in BM as compared to to IV-only transplanted animals. Overall, these results could be exploited for a clinical translation plan where the combined ICV+IV delivery could be particularly indicated for symptomatic children, who could not benefit from the standard HSC gene therapy approach, as shown in similar disease settings (refs di MLD e ALD trials). In these patients, an expectation of benefit, which could be greater than what could be achieved with a standard IV-only approach, could compensate the possible safety concerns associated to the novel intra-ventricular delivery

strategy. As far as the safety of intra-ventricular HSPC administration is concerned, our data included in the manuscript, as well as other preclinical and clinical data, may allow some drawing provisional comments. Firstly, we performed an extensive toxicological evaluation that was run in parallel to the efficacy assessment of all study animals. The evaluation included, after formal dose proof, a regular (every working day) observation for clinical signs/vitality, early detection of intercurrent deaths, hematology evaluation at dose proof and natural death/sacrifice, full necropsy at natural death/sacrifice, histopathological investigations on multiple tissues by an expert pathologist (outsourced) and in particular on the CNS. These analyses excluded tumor masses in the CNS or elsewhere in all study animals, including those in which the transduced HSPCs were administered ICV. As far as the risk of oncogenic transformation of the transduced HSPCs in the CNS or in hematopoietic organs is concerned , the safety of HSPC gene therapy has been extensively demonstrated in mice and patients, where follow up data reach almost 12 years post-transplant for the first children (Fumagalli et al., Lancet 2022, PMID: 35065785; Eichler et al., N Engl J Med. 2017, PMID: 28976817). In both the preclinical and clinical setting no adverse genotoxic events, nor skewing of the integration profile of the vector nor abnormal clonal dominance events nor actual tumors arising from the transplanted cells have been thus far observed when employing the same vector backbone here described (with the human PGK promoter). Obviously, we cannot exclude that transplantation of the transduced cells in the CNS could provide different stimuli and negatively affect this safety profile, but thus far our preclinical observation has excluded this event. In support of this, no safety concerns have thus far been reported when human HSPCs/their derivatives have been administered intra-ventricularly (NCT02254863).

Although the authors measured PPT1 activity in the brain, there were no other measures on cell engraftment in the brain. For example, staining for the PPT1 in various areas of the brain would have been informative.

Answer: According to the Reviewer's suggestion, we performed an immunohistochemistry study with an anti-human PPT1 antibody on the brain from representative mice transplanted with hPPT1.LV-transduced HSPCs. The results are shown in Fig. S3 of the revised manuscript.

The authors argued that storage in peripheral tissues might occur in CLN1. This hypothesis could be easily addressed by AF of the heart, at least.

***Answer*:** Unfortunately, we could not preserve heart tissue of adequate to perform a thorough examination in the experimental groups. This is due to the fact that animals were intracardially perfused with a syringe filled with PBS to remove blood before tissue dissection. However, the histopathological assessment performed on the other peripheral organs (liver, spleen, lung, bone marrow, sciatic nerve) confirmed the presence of foamy macrophages or vacuolated cells, indicative of a lysosomal pathology, in Ppt1 -/- untreated and mock transplanted mice. In fact, lysosomal storage diseases, including neuronal ceroid lipofuscinosis, can result in the accumulation of lipids in virtually any organ, with excessive

accumulation often seen in the lung, liver, brain, kidney, ocular tissues, heart, adrenal glands, hematopoietic tissues, and circulating lymphocytes.
A supplementary figure, showing these findings in peripheral organs (namely spleen and bone marrow, in supplementary Fig S6), has been added in the revised manuscript. A comment on this figure has been added in the results and in the discussion sections.

Major remarks

1. The DSS score figure 1 should have included a wt control group.
   Answer: A WT control group has been added in Figure 1 and, accordingly, also in Fig. S2B and Fig. 2A

2. Fig. 1F: lines with the statistical comparisons are not aligned to the bars.
   Answer: Actually, the lines of the statistical comparisons were correctly placed but the $Ppt1^{+/+}$ group was not visible on the graph since the AF value in $Ppt1^{+/+}$ mice was zero. This might have induced in a wrong interpretation. For this reason, we specified in the figure legend that "*The histograms of $Ppt1^{+/+}$ group are not visible in the graph as the AF signal was zero in these animals*". To help identifying the groups in the statistical comparison, we revised Fig.1F and we added a black segment on the x axis in the graph, corresponding to the $Ppt1^{+/+}$ group.

3. Fig. 1H: the rescue of visual cortex thickness was not found as there is no statistically significant difference between treated and untreated mice.
   Answer: We thank you the Reviewer for highlighting this aspect. The text has been revised accordingly.

4. Fig. 2: the labeling of the y axis is switched: the VCN should have the logarithmic scale and the enzyme activity the non-logarithmic scale.
   Answer: We confirm that the labeling of the y axis shown in Fig.2 is correct. Both the left and right y axes are displayed in non-logarithmic scale. However, the values shown on the left y axis (corresponding to the VCN) range between 0 and 20, whereas the values shown on the right y axis (corresponding to the enzymatic activity) range between 0 and 30,000 nmol/mg/h.

5. Fig. 2: on survival, DSS, limb stiffness and rotarod, there is no significant difference between IV and IV+ICV. Therefore, I do not understand how the multivariate analysis can show a statistical difference and even if that is the case, I wonder about the clinical relevance of this multivariate analysis.
   Answer: Even though we did not highlight any statistical significant difference between IV and IV+ICV in the single tests (namely DSS, limb stiffness and rotarod), we observed a slightly better performance of the IV+ICV as compared to other groups. To verify whether the performances in each single test were indicative of an overall better clinical condition of the ICV+IV animals, we repeated the analysis by comparing (through a multivariate analysis) at the same time all the measurements retrieved from

each animal. This analysis indeed highlighted a difference between the IV+ICV and the IV group. We interpreted this finding as a suggestion of a potential superiority of the IV+ICV protocol in achieving a clinical benefit. However, we hypothesized that the experimental setting (consisting in the transplantation at the pre-symptomatic stage) allowed an extensive rescue of the pathological phenotype independently from the route of administration. For this reason, we decided to repeat the experiment in a more challenging condition (i.e. transplantation at the late pre-symptomatic stage, few weeks before the expected disease onset). Overall, we believe that the clinical relevance of our results reside in the fact that ICV+IV determined a better disease outcome, as compared to the IV-only group, when the transplant was performed at the late pre-symptomatic stage, supporting the employment of the combinatorial transplantation strategy whenever a faster onset of the clinical benefit is needed.

6. Fig. 3A: by VCN and enzyme activity, authors are not evaluating cell engraftment. This will be evaluated by immunochemistry looking at the number of corrected cells in the brain.

Answer: According to the Reviewer's comment, we replaced the word "cell engraftment" with VCN in the text. We could not directly measure the number of corrected cells in the brain, as the transplanted cells were syngeneic with the recipient mice and there was no reporter gene expressed by the lentiviral vectors, further than the human PPT1. For this reason, we relied on VCN measurement and the immunohistochemistry for human PPT1 to estimate the efficiency of reconstitution in the brain.

7. Fig. 3C: AF signal in the 'mock tx' group appears to be reduced across all brain areas compared to the untreated group. The quantification shown in fig. 3D does not show a reduction, but I wonder whether representative images were selected.

Answer: We replaced the images shown in Fig.3C with pictures that were more representative of the results of the quantification.

8. AF immunofluorescence should be performed also in the mice receiving ICV + IV treatment after the onset of the symptoms.

Answer: AF analysis has been performed on the brain of mice transplanted at the symptomatic stage and sacrificed at end of study, >6 months after transplant. The results are shown in Fig. 5 of the revised manuscript.

9. The authors reported that transplanted mice evaluated for full necropsy. Did they look specifically for brain tumors? Were brain tumors ruled out and how?

Answer: No macroscopic findings suggestive of brain tumors were detected at necropsy. The histopathological assessment was performed in blind by an expert pathologist of an external CRO. The operator was not instructed to specifically look for brain tumors. However, no brain tumor or aberrant proliferation of cells was reported in any of the experimental groups. The analysis highlighted only cortical atrophy (to different extent depending on the treatment group),

Minor issues

1. Chromosomal location of PPT1 gene is not needed (page 3).

> ***Answer:*** Chromosomal location at pag.3 has been removed from the revised text.

2. The English requires attention. For example:

a. Page 4: '...HSC gene therapy my address this goal' rather than 'these indications'.

> ***Answer:*** The sentence at pag.4 has been adjusted according to the Reviewer's suggestion and the manuscript has been carefully revised for accuracy.

b. Page 4: 'proof of concept' rather than 'proof of applicability'.

> ***Answer:*** The sentence has been revised as follows: *"Here, we provide the first proof of concept of therapeutic efficacy…."*

c. Page 5: '6-8 week-old' rather than '6-8 weeks-old).

> ***Answer:*** The sentence at pag.5 has been adjusted.

d. Page 5 'administered with busulfan' rather than 'exposed to a busulfan-based'.

> ***Answer*****:** The sentence has been rephrased as follows: "*Ppt1-/- mice were administered with busulfan (as myeloablative conditioning)…"*

e. What is 'dose proof'?

> ***Answer:*** The term 'dose proof', derived from Good Laboratory Practices, indicates the evidence of actual and accurate dosing of each study animal, which could be documented as engraftment of transplanted HSPCs in hematopoietic organs. We have added this explanation in the revised manuscript: *"Dose proof, defined as the evidence of actual engraftment of transplanted HSPCs in hematopoietic organs, was obtained 35-42 days after transplant by demonstrating high vector copy number (VCN) in the PB of transplanted animals (Fig. 2B)".*

3. Please do not use 'PBC' acronym for 'poor body condition' as 'PBC' is typically used for peripheral blood cells'.

> ***Answer:*** The 'PBC' acronym was removed in the revised version of the manuscript.

4. Check that figures are numbered in the order they appear in the text: Fig. 5C is cited before 5B; Fig. S3 is cited for the first time on page 11 (after fig. S4).

> ***Answer:*** Following the Reviewer's suggestion, we adjusted Figure 5 to reflect the order in which figures are cited in the main text. Moreover, we reorganized the text, Figure 3 and Figure S4 (now Figure S5 in the revised manuscript) to make sure the figures are cited in a sequential order.

18th Nov 2022

Dear Dr. Biffi,

Thank you for the submission of your manuscript to EMBO Molecular Medicine, and please accept my apologies for the delay in getting back to you, which is due to the fact that one referee needed more time to complete his/her review. As you will see from the referee reports below, both referees acknowledge the improvements of the revised manuscript but also raise some concerns that should be addressed in additional and final round of revision. Referee #1 suggestions should be addressed by discussion in the text, while the referee #2 concern should be addressed by performing appropriate statistical tests to support the conclusion that combined IV + ICV delivery outperforms IV delivery. Addressing the reviewers' concerns in full will be necessary for further considering the manuscript in our journal, and acceptance of the manuscript will entail a third round of review.

In addition to addressing the referees' comments please amend the following:

1) Authors: E-mail correspondence to Rajesh Kumar, Valentina Poletti and Eleonora Cavalca could not be delivered. Please update their e-mail addresses and make sure to enter correct e-mail addresses for all authors in our submission system.
2) In the main manuscript file, please do the following:
- Correct/answer the track changes suggested by our data editors by working from the attached document.
- Remove list of abbreviations.
- Add callouts for Figure 2H.
- Move all supplementary figure legends to Appendix and leave only main figure legends.
- If no data are deposited in a public repository Data availability statement should only contain the sentence "This study includes no data deposited in external repositories". Please correct.
- Please rename "Competing Interest" to "Disclosure Statement & Competing Interests" and move it after the "Acknowledgements". We updated our journal's competing interests policy in January 2022 and request authors to consider both actual and perceived competing interests. Please review the policy https://www.embopress.org/competing-interests and update your competing interests if necessary.
- Author contributions: Please remove it from the manuscript and specify author contributions in our submission system. CRediT has replaced the traditional author contributions section because it offers a systematic machine-readable author contributions format that allows for more effective research assessment. You are encouraged to use the free text boxes beneath each contributing author's name to add specific details on the author's contribution. More information is available in our guide to authors:
https://www.embopress.org/page/journal/17574684/authorguide#authorshipguidelines
- Correct the reference citation in the text and reference list. In the text, a reference should be cited by author and year of publication. Include a space between a word and the opening parenthesis of the reference that follows. In the reference list, citations should be listed in alphabetical order. Where there are more than 10 authors on a paper, 10 will be listed, followed by "et al.". Please check "Author Guidelines" for more information.
https://www.embopress.org/page/journal/17574684/authorguide#referencesformat
3) Appendix: Please merge all supplementary figures and tables into a PDF file with a table of content on the title page and figure legends added below each figure. Rename all supplementary figures and tables to "Appendix Figure S1" etc and "Appendix Table S1" etc and update their callouts in the main manuscript text.
4) The Paper Explained: Please provide "The Paper Explained" and add it to the main manuscript text. Please check "Author Guidelines" for more information. https://www.embopress.org/page/journal/17574684/authorguide#researcharticleguide
5) Synopsis: Every published paper now includes a 'Synopsis' to further enhance discoverability. Synopses are displayed on the journal webpage and are freely accessible to all readers. They include separate synopsis image and synopsis text.
- Synopsis image: Please provide a striking image or visual abstract as a high-resolution jpeg file 550 px-wide x (250-400)-px high to illustrate your article.
- Synopsis text: Please provide a short standfirst (maximum of 300 characters, including space) as well as 2-5 one sentence bullet points that summarise the paper as a .doc file. Please write the bullet points to summarise the key NEW findings. They should be designed to be complementary to the abstract - i.e. not repeat the same text. We encourage inclusion of key acronyms and quantitative information (maximum of 30 words / bullet point). Please use the passive voice.
- Please check your synopsis text and image before submission with your revised manuscript. Please be aware that in the proof stage minor corrections only are allowed (e.g., typos).
6) For more information: Please remove data availability information. This space should be used to list relevant web links for further consultation by our readers. Could you identify some relevant ones and provide such information as well? Some examples are patient associations, relevant databases, OMIM/proteins/genes links, author's websites, etc...
7) Source data: We encourage you to include the source data for figure panels that show essential data. Numerical data should be provided as individual .xls or .csv files (including a tab describing the data). For blots or microscopy, uncropped images should be submitted (using a zip archive if multiple images need to be supplied for one panel). Please check "Author Guidelines" for more information. https://www.embopress.org/page/journal/17574684/authorguide#sourcedata
8) As part of the EMBO Publications transparent editorial process initiative (see our Editorial at http://embomolmed.embopress.org/content/2/9/329), EMBO Molecular Medicine will publish online a Review Process File (RPF)

to accompany accepted manuscripts. This file will be published in conjunction with your paper and will include the anonymous referee reports, your point-by-point response and all pertinent correspondence relating to the manuscript. Let us know whether you agree with the publication of the RPF and as here, if you want to remove or not any figures from it prior to publication. Please note that the Authors checklist will be published at the end of the RPF.
9) Please provide a point-by-point letter INCLUDING my comments as well as the reviewer's reports and your detailed responses (as Word file).

I look forward to reading a new revised version of your manuscript as soon as possible.

Yours sincerely,

Zeljko Durdevic

Zeljko Durdevic
Editor
EMBO Molecular Medicine

***** Reviewer's comments *****

Referee #1 (Comments on Novelty/Model System for Author):

The mouse model is essential for this work

Referee #1 (Remarks for Author):

The authors have addressed the points and I recommend publication. I would like to point out some remaining remarks which they might want to include in the final version:

ad point 1, genotoxicity:
It cannot be excluded that the route of administration will affect safety with respect to genotoxicity, as the immune system has a protective role in LV integration-mediated tumorigenesis, and this may be different for the periphery and the CNS.

ad point 2: VCN in CNS:
Authors did not comment on higher (circa 1 order of magnitude) VCN compared to others (e.g. Gleitz et al 2018 VCN in CNS 0.04, Miwa et al 2020 VCN in CNS 0.04).
This cannot be explained by a 2-fold higher VCN in PB.
Also note that after IV, some mice have a VCN in brain of 1 or 2, meaning that there is a lentiviral copy per each and every single cell in brain. I suspect that this may relate to differential methods to determine VCN, but I think it could be worthwhile to make a comment on this in the manuscript.

Referee #2 (Comments on Novelty/Model System for Author):

The authors should present statistical comaprison between IV and IV+ICV groups in survival, DSS, limb stifness, rotarod, etc.

Referee #2 (Remarks for Author):

Pevani and colleagues addressed most but one of the issues previously raised. Their main conclusion is that 'combinatorial transplantation of transduced HSPCs intravenously and ICV results in the most robust therapeutic benefit among the tested approaches, particularly on symptomatic animals'. While the data show that IV or IV+ICV results in phenotype improvements in the ppt1-/- mice, the data do not support superiority of IV+ICV over IV. The statistical analyses showing such difference are not shown.

Point by point reply to Referees' suggestions and comments

Referee #1 (Comments on Novelty/Model System for Author):
The mouse model is essential for this work

Referee #1 (Remarks for Author):
The authors have addressed the points and I recommend publication. I would like to point out some remaining remarks which they might want to include in the final version:

ad point 1, genotoxicity:
It cannot be excluded that the route of administration will affect safety with respect to genotoxicity, as the immune system has a protective role in LV integration-mediated tumorigenesis, and this may be different for the periphery and the CNS.

Answer: we would respectfully note that genotoxicity is mostly dependent on the intrinsic features (regulatory elements and integration site preference in the genome) of the LV construct, rather than the route of administration. However, in the path to clinical development, we agree with the Reviewer that a full in-depth toxicological evaluation, including potential genotoxic events, should be performed. For this reason, we added the following sentence in the Discussion section of the revised manuscript: *"To further support the safety of the intra-CNS transplantation of LV-transduced HSPCs, additional dedicated studies will be conducted on human HSPCs during the path to clinical translation".*

ad point 2: VCN in CNS:
Authors did not comment on higher (circa 1 order of magnitude) VCN compared to others (e.g. Gleitz et al 2018 VCN in CNS 0.04, Miwa et al 2020 VCN in CNS 0.04). This cannot be explained by a 2-fold higher VCN in PB. Also note that after IV, some mice have a VCN in brain of 1 or 2, meaning that there is a lentiviral copy per each and every single cell in brain. I suspect that this may relate to differential methods to determine VCN, but I think it could be worthwhile to make a comment on this in the manuscript.

Answer: as shown in Fig. 2A, our protocol of murine HSPC transduction is highly efficient and results in VCNs of ~9 on average in vitro, in the liquid culture as well as in CFCs. Differently from other studies (e.g. Miwa et al. 2020, PMID: 32631737), in our work we applied a pre-transplant conditioning regimen (based on busulfan administration) that allows achieving higher donor-cell chimerism in the brain and spinal cord, as compared to lethal irradiation (see Capotondo et al. 2012, PMID: 22923692; Capotondo, Milazzo et al. 2017, PMID: 29226242). In fact, as shown in Fig. 1C of the manuscript, in our transplant setting, IV administration of LV-transduced HSPCs leads to ~70% and ~87% donor-cell chimerism (measured by flow cytometry) in the peripheral blood and in the bone marrow, respectively. In the brain we could retrieve ~60% donor-cell chimerism in the CD11b+ microglia/macrophage population (measured by flow cytometry). Considering these results that highlight the high donor-cell chimerism achieved in target tissues in our transplant setting, we concluded that the high VCN retrieved in the BM (Fig. 3C), brain and spinal cord (Fig. 3A) does not necessarily implies that there is every single cell in the tissue carrying a copy of the lentiviral vector genome, but rather that in these tissues we achieved a high donor-cell chimerism with the progeny of the transplanted HSPCs carrying several copies of the LV in their genome. Accordingly, we added the following sentence in the Discussion session of the revised manuscript: "…we reported supra-physiological levels of Ppt1 enzymatic activity in the brain and spinal cord of the transplanted mice (~3.5-fold higher than the WT or WT HSPC transplanted animals). *This result was paralleled by high VCN retrieved in the CNS, further confirming that busulfan conditioning is instrumental to increase the efficiency of brain repopulation by the progeny of the transplanted HSPCs*."

Referee #2 (Comments on Novelty/Model System for Author):

The authors should present statistical comparison between IV and IV+ICV groups in survival, DSS, limb stifness, rotarod, etc.

Referee #2 (Remarks for Author):

Pevani and colleagues addressed most but one of the issues previously raised. Their main conclusion is that 'combinatorial transplantation of transduced HSPCs intravenously and ICV results in the most robust therapeutic benefit among the tested approaches, particularly on symptomatic animals'. While the data show that IV or IV+ICV results in phenotype improvements in the ppt1-/- mice, the data do not support superiority of IV+ICV over IV. The statistical analyses showing such difference are not shown.

Answer: following reviewer's suggestion, we added an Appendix Table S4 in the revised version of the manuscript showing the result of the statistical analyses performed on the longitudinal assessment of the behavioral parameters (namely DSS and rotarod). We would like to underline that the statistical test could be performed only for a specific time-range (specified in the table) since for some groups more than 50% of animals were missing values due to ICDs. Accordingly, we have decided to correct some sentences (listed below) in the Discussion session and in the Abstract to reflect more precisely the results obtained in this work.
Discussion: *"Notably, neuroinflammation, measured by signal intensity of the CD68 marker, was more substantially reduced in ICV+IV treated mice compared to ICV-only recipients, suggesting a synergistic neuroprotective effect of the combined transplantation modality. These findings and the additive benefit of the combined ICV+IV HSPC gene therapy approach were supported by the PCAs and the multiparametric statistical analyses performed on all histological as well as all behavioral analyses. Indeed, at the behavioral level, the ICV+IV group displayed a statistically superior therapeutic potential as compared to the IV- and ICV-only approaches (from 350 days of age), whereas at the histological level the ICV+IV was similar to the IV-only group and both approaches displayed a statistical superior benefit as compared to the ICV-only group".*
Abstract: *"…iv) the combinatorial transplantation of transduced HSPCs intravenously and ICV results in a robust therapeutic benefit, particularly on symptomatic animals".*

We are pleased to inform you that your manuscript is accepted for publication and is now being sent to our publisher to be included in the next available issue of EMBO Molecular Medicine.

***** Reviewer's comments *****

Referee #2 (Remarks for Author):

Pevani and colleagues have addressed the remaining concern about comparison between IV+ICV and IV treatment groups.

EMBO Press Author Checklist

Corresponding Author Name: Alessandra Biffi

Journal Submitted to: EMBO Mol Med
Manuscript Number: EMM-2022-15968

USEFUL LINKS FOR COMPLETING THIS FORM
The EMBO Journal - Author Guidelines
EMBO Reports - Author Guidelines
Molecular Systems Biology - Author Guidelines
EMBO Molecular Medicine - Author Guidelines

Reporting Checklist for Life Science Articles (updated January 2022)
This checklist is adapted from Materials Design Analysis Reporting (MDAR) Checklist for Authors. MDAR establishes a minimum set of requirements in transparent reporting in the life sciences (see Statement of Task: 10.31222/osf.io/9sm4x). Please follow the journal's guidelines in preparing your manuscript.
Please note that a copy of this checklist will be published alongside your article.

Abridged guidelines for figures
1. Data
The data shown in figures should satisfy the following conditions:
- the data were obtained and processed according to the field's best practice and are presented to reflect the results of the experiments in an accurate and unbiased manner.
- ideally, figure panels should include only measurements that are directly comparable to each other and obtained with the same assay.
- plots include clearly labeled error bars for independent experiments and sample sizes. Unless justified, error bars should not be shown for technical replicates.
- if n<5, the individual data points from each experiment should be plotted. Any statistical test employed should be justified.
- Source Data should be included to report the data underlying figures according to the guidelines set out in the authorship guidelines on Data Presentation.

2. Captions
Each figure caption should contain the following information, for each panel where they are relevant:
- a specification of the experimental system investigated (eg cell line, species name).
- the assay(s) and method(s) used to carry out the reported observations and measurements.
- an explicit mention of the biological and chemical entity(ies) that are being measured.
- an explicit mention of the biological and chemical entity(ies) that are altered/varied/perturbed in a controlled manner.
- the exact sample size (n) for each experimental group/condition, given as a number, not a range;
- a description of the sample collection allowing the reader to understand whether the samples represent technical or biological replicates (including how many animals, litters, cultures, etc.).
- a statement of how many times the experiment shown was independently replicated in the laboratory.
- definitions of statistical methods and measures:
  - common tests, such as t-test (please specify whether paired vs. unpaired), simple χ2 tests, Wilcoxon and Mann-Whitney tests, can be unambiguously identified by name only, but more complex techniques should be described in the methods section;
  - are tests one-sided or two-sided?
  - are there adjustments for multiple comparisons?
  - exact statistical test results, e.g., P values = x but not P values < x;
  - definition of 'center values' as median or average;
  - definition of error bars as s.d. or s.e.m.

Please complete ALL of the questions below.
Select "Not Applicable" only when the requested information is not relevant for your study.

Materials

Newly Created Materials	Information included in the manuscript?	In which section is the information available? (Reagents and Tools Table, Materials and Methods, Figures, Data Availability Section)
New materials and reagents need to be available; do any restrictions apply?	Yes	

Antibodies	Information included in the manuscript?	In which section is the information available? (Reagents and Tools Table, Materials and Methods, Figures, Data Availability Section)
For **antibodies** provide the following information:<br>- Commercial antibodies: RRID (if possible) or supplier name, catalogue number and or/clone number<br>- Non-commercial: RRID or citation	Yes	Materials and Methods

DNA and RNA sequences	Information included in the manuscript?	In which section is the information available? (Reagents and Tools Table, Materials and Methods, Figures, Data Availability Section)
**Short novel DNA or RNA including primers, probes**: provide the sequences.	Yes	Data Availability Section

Cell materials	Information included in the manuscript?	In which section is the information available? (Reagents and Tools Table, Materials and Methods, Figures, Data Availability Section)
**Cell lines:** Provide species information, strain. Provide accession number in repository **OR** supplier name, catalog number, clone number, and/**OR** RRID.	Not Applicable	
**Primary cultures:** Provide species, strain, sex of origin, genetic modification status.	Yes	Materials and Methods
Report if the cell lines were recently **authenticated** (e.g., by STR profiling) and tested for mycoplasma contamination.	Not Applicable	

Experimental animals	Information included in the manuscript?	In which section is the information available? (Reagents and Tools Table, Materials and Methods, Figures, Data Availability Section)
**Laboratory animals or Model organisms:** Provide species, strain, sex, age, genetic modification status. Provide accession number in repository **OR** supplier name, catalog number, clone number, **OR** RRID.	Yes	Materials and Methods
**Animal observed in or captured from the field:** Provide species, sex, and age where possible.	Not Applicable	
Please detail **housing and husbandry conditions**.	Yes	Materials and Methods

Plants and microbes	Information included in the manuscript?	In which section is the information available? (Reagents and Tools Table, Materials and Methods, Figures, Data Availability Section)
**Plants:** provide species and strain, ecotype and cultivar where relevant, unique accession number if available, and source (including location for collected wild specimens).	Not Applicable	
**Microbes:** provide species and strain, unique accession number if available, and source.	Not Applicable	

Human research participants	Information included in the manuscript?	In which section is the information available? (Reagents and Tools Table, Materials and Methods, Figures, Data Availability Section)
If collected and within the bounds of privacy constraints report on age, sex and gender or ethnicity for all study participants.	Not Applicable	

Core facilities	Information included in the manuscript?	In which section is the information available? (Reagents and Tools Table, Materials and Methods, Figures, Data Availability Section)
If your work benefited from core facilities, was their service mentioned in the acknowledgments section?	Yes	Acnkowledgements

Design

Study protocol	Information included in the manuscript?	In which section is the information available? (Reagents and Tools Table, Materials and Methods, Figures, Data Availability Section)
If study protocol has been **pre-registered, provide DOI in the manuscript**. For clinical trials, provide the trial registration number **OR** cite DOI.	Not Applicable	
Report the **clinical trial registration number** (at ClinicalTrials.gov or equivalent), where applicable.	Not Applicable	

Laboratory protocol	Information included in the manuscript?	In which section is the information available? (Reagents and Tools Table, Materials and Methods, Figures, Data Availability Section)
Provide DOI OR other citation details if **external detailed step-by-step protocols** are available.	Not Applicable	

Experimental study design and statistics	Information included in the manuscript?	In which section is the information available? (Reagents and Tools Table, Materials and Methods, Figures, Data Availability Section)
Include a statement about **sample size** estimate even if no statistical methods were used.	Yes	Materials and Methods, Figures
Were any steps taken to minimize the effects of subjective bias when allocating animals/samples to treatment (e.g. **randomization procedure**)? If yes, have they been described?	Yes	Materials and Methods
Include a statement about **blinding** even if no blinding was done.	Yes	Materials and Methods
Describe **inclusion/exclusion criteria** if samples or animals were excluded from the analysis. Were the criteria pre-established?<br><br>If sample or data points were omitted from analysis, report if this was due to attrition or intentional exclusion and provide justification.	Yes	Materials and Methods
For every figure, are **statistical tests** justified as appropriate? Do the data meet the assumptions of the tests (e.g., normal distribution)? Describe any methods used to assess it. Is there an estimate of variation within each group of data? Is the variance similar between the groups that are being statistically compared?	Yes	Materials and Methods, Figures

Sample definition and in-laboratory replication	Information included in the manuscript?	In which section is the information available? (Reagents and Tools Table, Materials and Methods, Figures, Data Availability Section)
In the figure legends: state number of times the experiment was **replicated** in laboratory.	Select response	
In the figure legends: define whether data describe **technical or biological replicates**.	Yes	

Ethics

Ethics	Information included in the manuscript?	In which section is the information available? (Reagents and Tools Table, Materials and Methods, Figures, Data Availability Section)
Studies involving **human participants**: State details of **authority granting ethics approval** (IRB or equivalent committee(s), provide reference number for approval.	Not Applicable	
Studies involving **human participants**: Include a statement confirming that **informed consent** was obtained from all subjects and that the experiments conformed to the principles set out in the WMA Declaration of Helsinki and the Department of Health and Human Services Belmont Report.	Not Applicable	
Studies involving **human participants:** For publication of **patient photos**, include a statement confirming that consent to publish was obtained.	Not Applicable	
Studies involving experimental **animals**: State details of **authority granting ethics approval** (IRB or equivalent committee(s), provide reference number for approval. Include a statement of compliance with ethical regulations.	Yes	
Studies involving **specimen and field samples:** State if relevant **permits** obtained, provide details of authority approving study; if none were required, explain why.	Not Applicable	

Dual Use Research of Concern (DURC)	Information included in the manuscript?	In which section is the information available? (Reagents and Tools Table, Materials and Methods, Figures, Data Availability Section)
Could your study fall under dual use research restrictions? Please check biosecurity documents and list of **select agents and toxins** (CDC): https://www.selectagents.gov/sat/list.htm	Not Applicable	
If you used a select agent, is the security level of the lab appropriate and reported in the manuscript?	Not Applicable	
If a study is subject to dual use research of concern regulations, is the name of the **authority granting approval and reference number** for the regulatory approval provided in the manuscript?	Not Applicable	

Reporting

The MDAR framework recommends adoption of discipline-specific guidelines, established and endorsed through community initiatives. Journals have their own policy about requiring specific guidelines and recommendations to complement MDAR.

Adherence to community standards	Information included in the manuscript?	In which section is the information available? (Reagents and Tools Table, Materials and Methods, Figures, Data Availability Section)
State if relevant guidelines or checklists (e.g., **ICMJE, MIBBI, ARRIVE, PRISMA**) have been followed or provided.	Yes	Materials and Methods
For **tumor marker prognostic studies**, we recommend that you follow the **REMARK** reporting guidelines (see link list at top right). See author guidelines, under 'Reporting Guidelines'. Please confirm you have followed these guidelines.	Not Applicable	
For **phase II and III randomized controlled trials**, please refer to the **CONSORT** flow diagram (see link list at top right) and submit the CONSORT checklist (see link list at top right) with your submission. See author guidelines, under 'Reporting Guidelines'. Please confirm you have submitted this list.	Not Applicable	

Data Availability

Data availability	Information included in the manuscript?	In which section is the information available? (Reagents and Tools Table, Materials and Methods, Figures, Data Availability Section)
Have **primary datasets** been deposited according to the journal's guidelines (see 'Data Deposition' section) and the respective accession numbers provided in the Data Availability Section?	Not Applicable	
Were **human clinical and genomic datasets** deposited in a public access-controlled repository in accordance to ethical obligations to the patients and to the applicable consent agreement?	Not Applicable	
Are **computational models** that are central and integral to a study available without restrictions in a machine-readable form? Were the relevant accession numbers or links provided?	Not Applicable	
If publicly available data were reused, provide the respective **data citations in the reference list**.	Not Applicable	
